# A reparative neutrophil subpopulation accelerates spinal cord regeneration in zebrafish by controlling macrophage inflammation via Il-4

**DOI:** 10.1186/s12974-026-03878-0

**Published:** 2026-05-26

**Authors:** Xiaobo Tian 田晓波, Alberto Docampo-Seara, Kim Heilemann, Friederike Kessel, Daniela Zöller, Anja Bretschneider, Thomas Becker, Catherina G. Becker

**Affiliations:** 1 Center for Regenerative Therapies Dresden at the TU Dresden, Fetscherstraße 105, Dresden, 01307 Germany; 2 Institute for Neuroscience and Cardiovascular Research, University of Edinburgh Medical School: Biomedical Sciences, Edinburgh, EH16 4SB UK; 3 Cluster of Excellence Physics of Life, TU Dresden, Dresden, 01062 Germany

**Keywords:** Innate immune response, Spinal cord regeneration, Il-1β, Axon regrowth, Behavioural recovery, Macrophages/microglia, Diphenyleneiodonium, Nitroreductase ablation

## Abstract

In mammals, a dysregulated immune response is detrimental to spinal cord repair. In zebrafish, which are capable of spinal cord regeneration, the immune response promotes regeneration. Neutrophils are the first immune cells to arrive at a spinal cord injury site, but their role in successful regeneration is not fully understood. Here we show that ablating neutrophils, including a subpopulation that expresses the cytokine *il4*, increases expression of *il1b* (coding for Il-1β) mainly in macrophages/microglia and delays anatomical and functional recovery after a spinal cord injury in larval zebrafish. Experimentally reducing Il-1β levels rescues axonal regeneration. Disruption of *il4* mimics the detrimental effect of neutrophil ablation for axonal regeneration and this is also rescued by reducing Il-1β levels. Moreover, after ablation of neutrophils, axonal regeneration and *il1b* expression levels are both rescued by over-expression of *il4*. Hence, after spinal cord injury, a pro-regenerative neutrophil subpopulation accelerates spinal cord regeneration in larval zebrafish by controlling expression of *il1b* mainly in macrophages/microglia. For this neutrophil action, *il4* expression is necessary and sufficient.

**Supplementary Information:**

The online version contains supplementary material available at https://doi.org/10.1186/s12974-026-03878-0.

## Introduction

Spinal cord injury leads to damage of axonal tracts that is not repaired in most mammals, including humans, often leading to permanent disability [1, 2]. In contrast, larval and adult zebrafish regrow axons and recover locomotion after complete spinal cord transection [3–5]. The immune reaction after injury in mammals is protracted, causing an inflammatory environment that is not conducive to regeneration [6–8]. Neutrophils and macrophages/microglia are the major cell types of the innate immune response that persist at the injury site in mammals [9]. 

In both, regenerating and non-regenerating systems, neutrophils are the first immune cells that arrive at a spinal cord injury site [10, 11]. Neutrophils negatively influence the balance of pro- and anti-inflammatory cytokines expressed by macrophages and are generally found to be detrimental for repair [12–16]. However, a sub-population of neutrophils has recently been discovered that promotes regeneration of the optic nerve in mice by secreting several growth factors [17]. Understanding neutrophil phenotypes is crucial also for therapeutic approaches. For example, transplantation of neural stem cells to repair the spinal cord is aided by removal of neutrophils [18]. Influencing the neutrophil phenotype towards a more pro-regenerative one or preserving pro-regenerative neutrophils could aid transplantation experiments.

In the highly accessible larval zebrafish model of spinal cord injury [19], neutrophils also exhibit a strong accumulation in a spinal cord injury site within minutes after spinal cord injury [20–22]. Preventing neutrophils from reaching the injury site during early post-lesion time points impairs axonal regrowth and behavioural recovery, whereas accelerating neutrophil resolution improves regeneration, suggesting positive roles for neutrophils in the initial phase of regeneration, but not during later phases in regenerating zebrafish [20]. In an experimental situation in which macrophages are missing, the increased number of remaining neutrophils contribute to an increased expression of the pro-inflammatory cytokine *il1b* [22]. High levels of *il1b* expression inhibit spinal cord regeneration in larval zebrafish [23]. Removing the neutrophils partially rescues reduced axon regrowth in the absence of macrophages, indicating that macrophages can suppress an anti-regenerative phenotype in neutrophils [22]. In addition, neutrophils may favourably influence the mechanical properties of the lesion tissue [21]. However, whether subpopulations of neutrophils exist that may be pro- or anti-regenerative and how these may signal to other cells in the injury site is not known.

Here we characterize a substantial sub-population of neutrophils that expresses the cytokine *il4* and supports regeneration. Ablating neutrophils delays regeneration in larval zebrafish and leads to upregulation of *il1b* in macrophages. Reducing elevated levels of Il-1β or over-expression of *il4* fully rescues axonal regeneration after pan-neutrophil ablation. Thus, we identify a pro-regenerative sub-population of neutrophils that accelerates regeneration by reducing *il1b* expression in macrophages/microglia via Il-4.

## Results

### Selective ablation of neutrophils delays spinal cord regeneration

Neutrophils arrive in the injury site within minutes, leading to an early peak of neutrophil invasion of the injury site at 4 h post-lesion (hpl) [22]. To establish a role of neutrophils in spinal cord regeneration in zebrafish, we decided to selectively remove these cells prior to injury and thus reduce neutrophil invasion of the injury site. To that aim, we used transgenic fish in which a fusion protein of the bacterial enzyme nitroreductase (NTR) and the fluorescent reporter mCherry was expressed under the control of the regulatory sequences of the neutrophil-specific gene *mpx* (*mpx:NTR-mCherry*; see Material and Methods). Animals were pre-incubated with the prodrug Metronidazole (MTZ), which is turned into a cytotoxic compound in cells expressing NTR, 8 h prior to the time point of spinal cord injury at 3 days post-fertilization (dpf). To monitor the effectiveness of this treatment, we determined cell death in the caudal hematopoietic tissue (CHT), which is ventral to the prospective injury site and the major place of origin for immune cells that invade the lesion site [22]. We found co-labelling of mCherry and the cell death indicator acridine orange in the CHT in 89.7% of all mCherry-expressing cells in unlesioned animals at 3 dpf, indicating that these were dead cells (Suppl. Figure 1 A-C). Double-labelling of macrophages with an anti-Mfap4 antibody indicated that the number of macrophages in the CHT was not changed by neutrophil ablation and that some macrophages had engulfed mCherry-positive profiles, providing further evidence for successful ablation (Suppl. Figure 1D-F). Consequently, at 4 hpl, *mpx:NTR-mCherry*-positive neutrophils accumulated in the lesion site in control animals, but in MTZ-treated animals this was not the case. The sparse mCherry signal in the lesion site was associated only with macrophages (Suppl. Figure 1G-I). We confirmed successful reduction of neutrophil numbers in the lesion using an antibody to Mpx (Suppl. Figure 1 J, K).

**Fig. 1:**
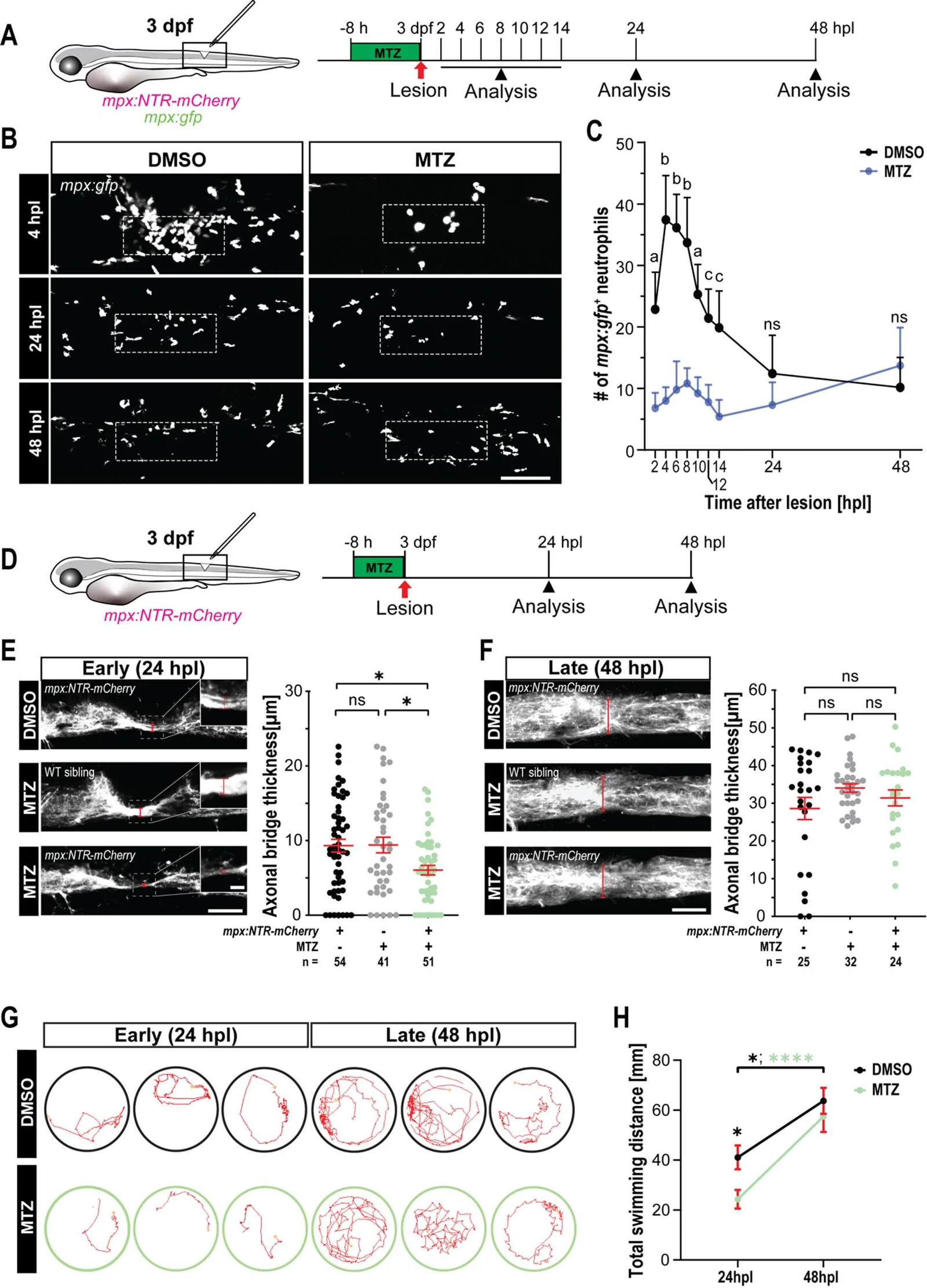
Ablation of neutrophils delays spinal cord repair. **A**: A schematic of a lateral view of a zebrafish larva indicating the spinal cord lesion (boxed) is shown. Rostral is left. Unless otherwise specified, this view is adopted throughout the article. The experimental timeline of nitroreductase/metronidazole mediated neutrophil ablation in *mpx:NTR-mCherry*; *mpx:gfp* larvae, corresponding to (B,C), is also shown. **B**, **C**: MTZ treatment significantly reduces neutrophil numbers from 2 to 14 hpl compared to DMSO controls. Dashed boxes mark the quantification area that is centered on the lesion. (Two-way ANOVA, interaction (F (8,108) = 14.54, *p* < 0.0001), time (F (8,108) = 12.45, p < 0.0001), and treatment (F (1,108) = 242.9,* p* < 0.0001). Tukey’s multiple comparisons test (DMSO vs. MTZ): a = *p* < 0.001, b = *p* < 0.0001, c = p < 0.01, ns indicates no significance. n DMSO = 7, n MTZ = 5). **D**: A schematic timeline for (E-H) is shown. **E**, **F**: Larvae that underwent neutrophil-ablation (*mpx:NTR-mCherry*^+^, MTZ^+^) show reduced axonal bridge thickness, as revealed by anti-acetylated tubulin labelling, at 24 hpl (E) but not at 48 hpl (F), compared to the indicated different control conditions. Dashed boxes mark the areas enlarged in the magnified views of the bridge. Red lines indicate the thickness of the regenerating spinal cord bridge measured for quantification (E: Kruskal-Wallis test, p = 0.0126. Dunn’s multiple comparisons test: DMSO (*mpx:NTR-mCherry*) vs. MTZ (*mpx:NTR-mCherry*), *p = 0.0229; MTZ (WT sibling) vs. MTZ (*mpx:NTR-mCherry*), *p = 0.0491. F: Kruskal-Wallis test, p = 0.7796; ns indicates no significance). **G**, **H**: Larvae that underwent neutrophil-ablation (MTZ) show reduced locomotor activity at 24 hpl, which recovered at 48 hpl, compared to control larvae (H, DMSO). Representative swimming behavior tracks of larvae at 24 and 48 hpl are shown (G). (Two-way ANOVA, interaction (F (1,188) = 1.507, *p* = 0.2211), time (F (1,188) = 29.08, p < 0.0001), and treatment (F (1,188) = 6.052, p = 0.0148). Tukey's multiple comparisons test: DMSO (24 hpl vs. 48 hpl), *p = 0.0189; MTZ (24 hpl vs. 48 hpl), ****p < 0.0001; 24 hpl (DMSO vs. MTZ), *p = 0.0480). Error bars show SEM. Scale bars: 100 μm (B), 50 μm (E, F), 10 μm (zoom-view of E). Images in (B, E and F) are shown as maximum intensity projections of Z-stacks encompassing the spinal cord region (*n* = 35 slices, step size = 1 μm)

For a time-course analysis, we crossed *mpx:NTR-mCherry* into a second neutrophil reporter line (*mpx: gfp)*. In this way, we avoided confounding mCherry debris, as the half-life of GFP was considerably shorter than that of mCherry in vivo [24, 25]. In control animals that had not been incubated with MTZ, we observed a sharp peak in neutrophil abundance in the injury site at 4 h in accordance with previous observations [22]. In contrast, in experimental animals, this peak was almost completely abolished. Low numbers of neutrophils were present in experimental animals for up to 24 hpl, at which time there was no difference to the low numbers of neutrophils in control animals anymore (Fig. 1A-C). Hence, we specifically ablated the early peak of neutrophil invasion in our experimental paradigm, but we cannot exclude that much lower numbers of neutrophils at later time points also contributed to regulation of the regenerative response.

To examine the consequences of neutrophil ablation for spinal cord regeneration, we analyzed axon regrowth, regenerative neurogenesis, and behavioral recovery. Only animals with complete transection of the spinal cord and the notochord left intact entered the experiment. Axon regrowth that reconnects the two spinal stumps was assessed by measuring the thickness of the axonal bundle in the injury site, as previously established [26]. This indicated a marked reduction in bridge thickness in neutrophil-ablated animals, compared to control groups at 24 hpl (35.2% reduction compared to transgenic animals without MTZ incubation; 35.8% reduction compared to wild type animals with MTZ incubation – controls were not different from each other). At 48 hpl, all groups showed comparable thickness of the bridge, indicating delayed axon regrowth in the absence of the neutrophil peak (Fig. 1D-F).

Similarly, regenerative neurogenesis, measured by EdU incorporation in motor neurons in *mnx1:gfp* reporter animals, as previously established [27, 28], indicated reduced numbers of newly generated motor neurons at 24 hpl compared to the above control groups (Suppl. Figure 2 A-C). At 48 hpl no difference was observed (Suppl. Figure 2D-E). Ongoing developmental neurogenesis in unlesioned animals was not affected at the corresponding time points, in line with absence of neutrophils during developmental neurogenesis.

Next, we measured the total distance swum after a vibration stimulus of animals at 24 and 48 hpl [23]. MTZ incubation did not detectably affect swimming distance in unlesioned animals (Suppl. Figure 3 A-C). In lesioned animals, the distance swum at 24 hpl was 40.8% reduced in MTZ treated *mpx:NTR-mCherry* animals, compared to DMSO-treated controls. At 48 hpl, there was no difference anymore, consistent with the dynamics of regeneration and neurogenesis (Fig. 1G, H). Hence, in the absence of the neutrophil peak, axon regrowth, regenerative neurogenesis, and behavioral recovery were delayed, indicating an important role of neutrophils in promoting the regeneration process. For an alternative approach to reduce presence of neutrophils in the lesion, we blocked neutrophil infiltration of the lesion by inhibiting NADPH oxidase with diphenyleneiodonium chloride (DPI) in an 8 h drug treatment from 4 h before to 4 h after lesion [29]. This treatment successfully reduced neutrophil infiltration by 37.9% at 4 hpl, which was less efficient than neutrophil ablation. At 24 hpl, 20 h after wash-out, there was no difference in neutrophil number between control and DPI-treated larvae (Suppl. Figure 4 A-C). The number of Macrophages (labelled by the *mpeg1:mCherry* transgene) remained unaffected at 4 and 24 hpl (Suppl. Figure 4D, E). DPI treatment reduced the thickness of the axon bridge by 49.5% at 24 hpl and by 27.4% at 48 hpl. The reduction of axon regrowth at 24 hpl is in line with the effect of neutrophil ablation. However, the persistent effect at 48 hpl suggests additional effects of the drug on other cell types (Suppl. Figure 4 F-H).

### Ablation of neutrophils changes the inflammatory environment of the lesion site

We reasoned that the lack of neutrophils would affect the cytokine milieu in the lesion site. Therefore, we analyzed changes in immune system regulation by determining levels of expression of pro- (*il1b*,* tnfa*) and anti-inflammatory cytokines (*tgfb1a*,* tgfb3*) by qRT-PCR of lesion site-enriched trunk tissue (relative to unlesioned controls) at the peak of neutrophil invasion at 4 hpl, at 24 hpl, when regeneration phenotypes were observed after neutrophil ablation and at the end of our observation period at 48 hpl.

**Fig. 2:**
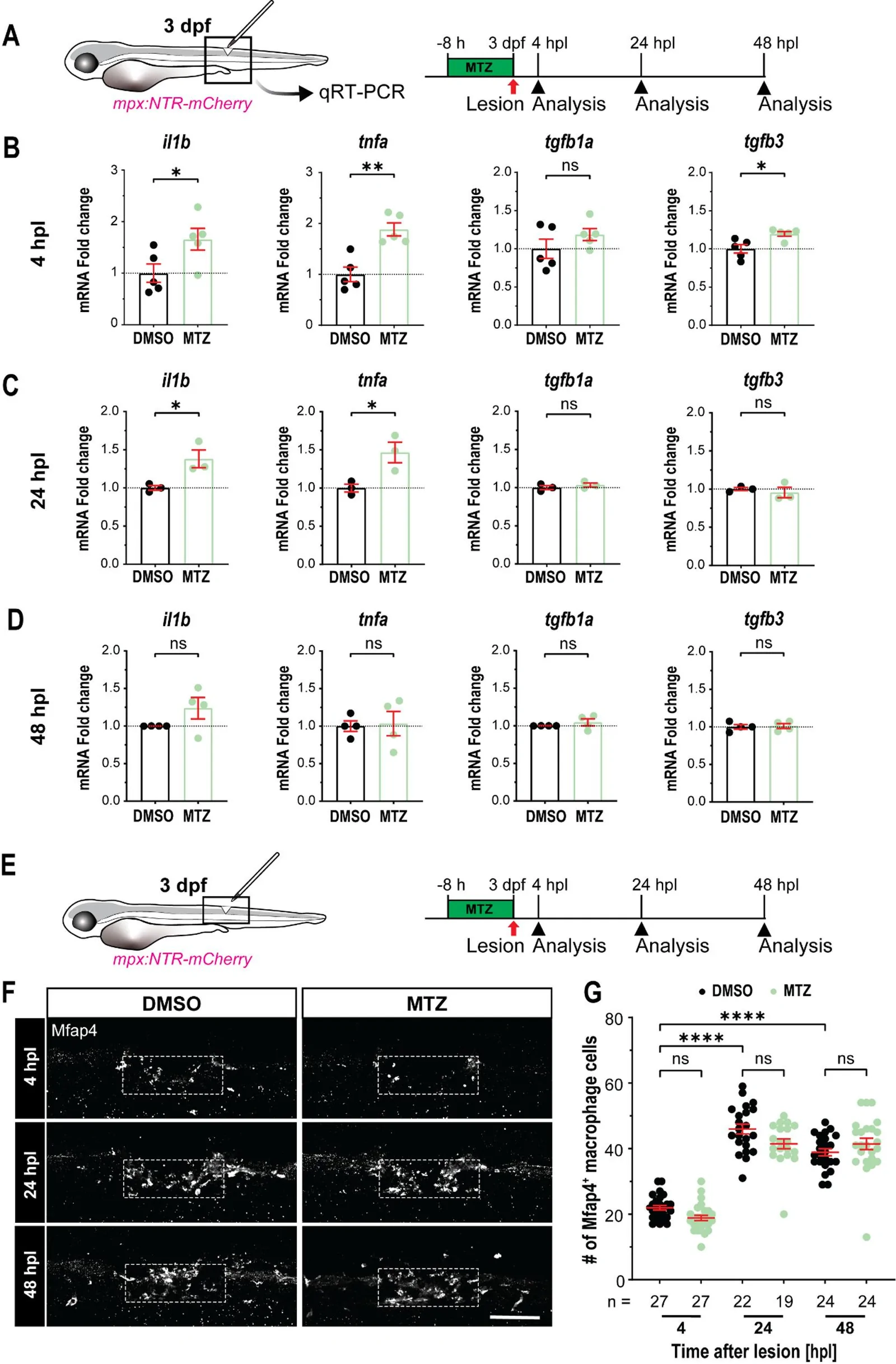
Neutrophil ablation leads to a transient increase in expression of pro-inflammatory cytokines in the lesion site. **A**: A schematic timeline of assessing pro-inflammatory (*il1b*, *tnfa*) and anti-inflammatory (*tgfb1a*, *tgfb3*) cytokines expression in mpx:NTR-mCherry larvae by qRT-PCR, corresponding to (B-D), is shown. **B**, **C**, **D**: Neutrophil ablation leads to a strong upregulation of il1b and tnfa at 4 and 24 hpl, but not at 48 hpl, with only a slight increase in *tgfb3* at 4 hpl (B). Each dot represents one biological replicate comprising 35 larvae. (Two-tailed unpaired t-test: at 4 hpl, *il1b* (*p = 0.0450), *tnfa* (**p = 0.0018), *tgfb3* (*p = 0.0202); at 24 hpl, il1b (*p = 0.0338), tnfa (*p = 0.0313); ns indicates no significance). **E**: A schematic timeline for (F, G) is shown. **F**, **G**: Neutrophil ablated larvae (MTZ) show no difference in in numbers of Mfap4^+^ macrophages compared to controls. Dashed boxes mark the quantification area that is centered on the lesion. (Two-way ANOVA, interaction (F (2,137) = 4.464, *p* = 0.0132), time (F (2,137) = 217.1, *p* < 0.0001), and treatment (F (1,137) = 2.774, *p* = 0.0981). Tukey’s multiple comparisons test: ****p < 0.0001, ns indicates no significance). Error bars show SEM. Scale bar: 100 μm (F). Images in F are shown as maximum intensity projections of Z-stacks encompassing the spinal cord region (*n *= 35 slices, step size = 1 μm)

In lesioned wild type animals, expression of pro-inflammatory cytokine *il1b* was unchanged by incubation with MTZ (Suppl. Figure 5 A, B). In contrast, in *mpx:NTR-mCherry* transgenic animals, *il1b* expression was increased by 65.9% at 4 hpl and 38% at 24 hpl, but not at 48 hpl after MTZ- treatment. Similarly, the pro-inflammatory cytokine *tnfa* was upregulated by 88.2% at 4 hpl and 46.7% at 24 hpl, but not anymore at 48 hpl in the absence of neutrophils. In contrast, anti-inflammatory cytokine *tgfb1a* showed no change in expression at any timepoint and anti-inflammatory cytokine *tgfb3* showed a 19.6% upregulation at 4 hpl, but no changes at 24 and 48 hpl (Fig. 2A-D). Numbers of Mfap4 immuno-positive macrophages in the injury site remained unchanged in MTZ-treated animals, compared to DMSO-treated controls at 4, 24 and 48 hpl (Fig. 2E-G). This indicated that macrophages could accumulate in the injury site without major neutrophil invasion and that macrophages and potentially other cell types in the lesion site environment showed increased expression of mainly pro-inflammatory cytokines, when neutrophils were ablated [23].

### Increased il1b expression delays axonal regeneration after neutrophil ablation

Increased levels of Il-1β have previously been shown to be detrimental to regeneration [22, 23, 30] and therefore, we aimed to rescue regeneration after neutrophil ablation by disrupting *il1b*. We used a dose-response approach with the caspase-1 inhibitor YVAD, an established drug to reduce Il-1β-signalling by inhibiting Caspase-1 dependent processing of the Il-1β protein in zebrafish larvae [22, 30]. YVAD treatment (50 µM) reduced expression of *il1b* (qRT-PCR and HCR) and *tnfa*, as also previously reported [22]. In addition, there was an increase in expression of *tgfb1a* (Suppl. Figure 6 A-D), possibly downstream of altered Il-1β-signaling [31].

**Fig. 3:**
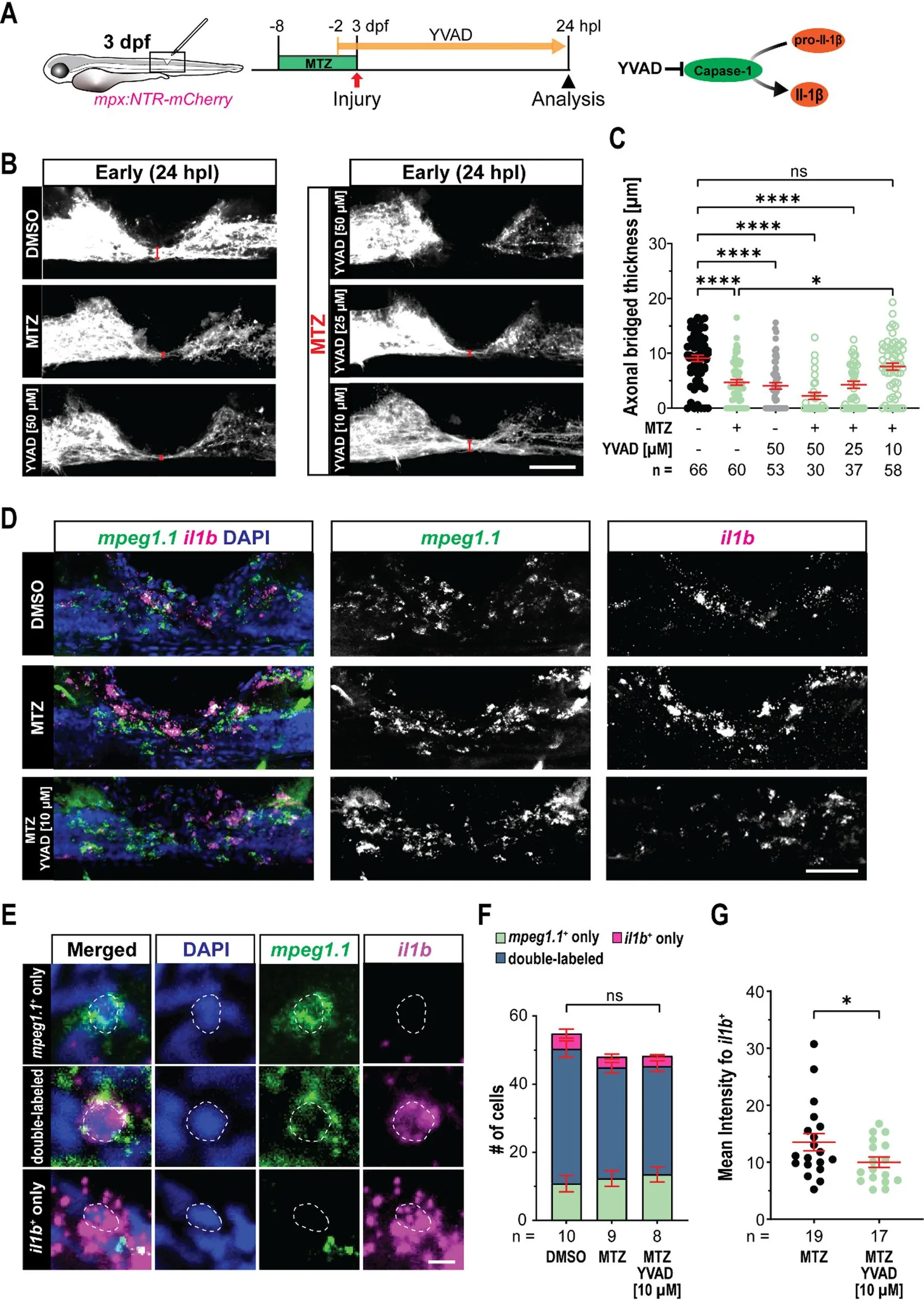
Low-dose inhibition of Il-1β processing restores spinal cord regeneration after neutrophil ablation. **A**: A schematic timeline of YVAD treatment in *mpx:NTR-mCherry* larvae for B-G is shown. **B**, **C**: 10 µM YVAD treatment restores axonal bridge thickness in larvae that underwent neutrophil ablation, compared to DMSO-treated controls (C). Images of spinal cord lesions labelled with anti-acetylated tubulin in different treatment groups at 24 hpl are shown (B). (Kruskal-Wallis test, *p* < 0.0001. Dunn’s multiple comparisons test: *p = 0.0131, ****p < 0.0001, ns indicates no significance). **D**, **E**: Images of *mpeg1.1* and *il1b* double-HCR in control (DMSO), neutrophil-ablated group (MTZ), and low-dose YVAD-treated neutrophil-ablated (MTZ, 10 µM YVAD) larvae at 24 hpl are shown (D). High-magnification views of individual *mpeg1.1*^+^ (green), *il1b*^+^ (magenta), and *mpeg1.1*^+^, *il1b*^+^ double-labeled cells from D. Dashed lines outline the corresponding cell nucleus (E). **F**: Neutrophil ablation does not reduce the number of *il1b-* expressing macrophages (*mpeg1.1*^+^, *il1b*^+^), compared with the DMSO control. This is not changed by YVAD treatment (Two-way ANOVA, interaction (F (4,74) = 2.365, *p* = 0.0606), treatment (F (2,74) = 1.517, *p* = 0.2261), and cell category (F (2,74) = 223.9, *p* < 0.0001), ns indicates no significance). **G**: Neutrophil ablation increases mean *il1b* fluorescence intensity, which is partially reduced by 10 µM YVAD treatment. (One-tailed Mann-Whitney test, *p = 0.0379). Error bars show SEM. Scale bar: 50 μm (B, D), 5 μm (E). Images are shown as maximum intensity projections of Z-stacks encompassing the spinal cord region (B: *n* = 35 slices, step size = 1 μm; D, E: *n* = 5 slices, step size = 1 μm)

The highest concentration of YVAD (50 µM) was not able to rescue the axonal bridging phenotype, but exacerbated the effect of neutrophil ablation. A 25 µM concentration did also not rescue the phenotype. In contrast, 10 µM concentration restored the thickness of the axon bridge to levels that were not different from those in untreated lesioned control animals. This indicates that the delayed regeneration in neutrophil-ablated animals could at least in part be explained by increased levels of *il1b* expression and/or dysregulation of other cytokines, such as *tnfa* or *tgfb1a* (Fig. 3A-C).

The observation that the highest YVAD concentration exacerbated regeneration could have been due to non-specific actions of the drug. Alternatively, it could have been because of low levels of Il-1β actually being needed for optimal regeneration. Previously, we have shown that disrupting Il-1β-signaling during early regeneration impairs regeneration, but not during late regeneration [22].

We therefore decided to disrupt the *il1b* gene in somatic mutants by injecting highly active CRISPR gRNAs into eggs, following a previously published protocol [30]. Gene disruption in somatic mutants was highly efficient, as indicated by the observation that a targeted restriction enzyme recognition site in the coding sequence became almost completely resistant to digestion, as shown by restriction fragment length polymorphism analysis (Suppl. Figure 7 A, B). We observed that disruption of *il1b* impaired axonal regeneration, compared to control gRNA-injected animals as expected [22], but did not rescue axon bridging in neutrophil-ablated larvae (Suppl. Figure 7 C-E). These observations were consistent with low levels of *il1b* expression being necessary for optimal regeneration, but exaggerated levels also being detrimental [22, 23, 30].

### Macrophages/Microglia are the major source of increased il1b expression after neutrophil ablation

To examine the likely cellular source of Il-1β in the lesion core, we re-examined previously published scRNA-seq data for unlesioned and 24 hpl larvae [27]. In both unlesioned and lesioned larvae, *il1b* was strongly expressed in neutrophils and macrophages. In contrast, *il4* was specific for neutrophils (Suppl. Figure 8 A, B). For a temporally and spatially resolved in vivo analysis, we counted *il1b*-positive neutrophil (*mpx:gfp*) and macrophage (*mpeg1:mCherry*) profiles in the same animals by HCR. In unlesioned animals, there were hardly any *il1b*^*+*^ profiles. At 6 hpl, we observed 30.3 *il1b*^*+*^ neutrophils and 7.7 macrophages per animals in the lesion. At 24 hpl, when axon regrowth was underway, we only observed 5.4 *il1b*^*+*^ neutrophils, but 33.9 *il1b*^*+*^ macrophages. Both cell types were strongly reduced in number again by 48 hpl (4.4 *il1b*^*+*^ neutrophils and 11.2 *il1b*^*+*^ macrophages; Suppl. Figure 8 C; D). Hence, there was a switch in the cell type that expressed *il1b* from neutrophils to macrophages during axon regrowth.

Next, we assessed *il1b *expression in control, MTZ-treated, and MTZ- and YVAD-treated animals in relation to *mpeg1.1*-expressing macrophages/microglia. We did not consider peripheral *il1b* expression in the skin [22]. This showed that the vast majority of *il1b*-expressing cells were *mpeg1.1*-positive during regeneration (between 89.8 and 91.4% of all *il1b*^+^ profiles at 24 hpl). About 75% of all macrophages in the injury site expressed *il1b*. There were no noticeable changes in these ratios between conditions. Therefore, we asked whether the intensity of labelling was reduced by the YVAD treatment. Indeed, the average labeling intensity for *il1b* after MTZ treatment was reduced by 21.15% by co-treatment with 10 µM YVAD (Fig. 3D-G). Hence, ablation of neutrophils leads to increased expression of *il1b*, mainly in macrophages/microglia and this could be reduced again by YVAD treatment.

### A subpopulation of neutrophils may signal via Il-4 at the injury site

**Fig. 4:**
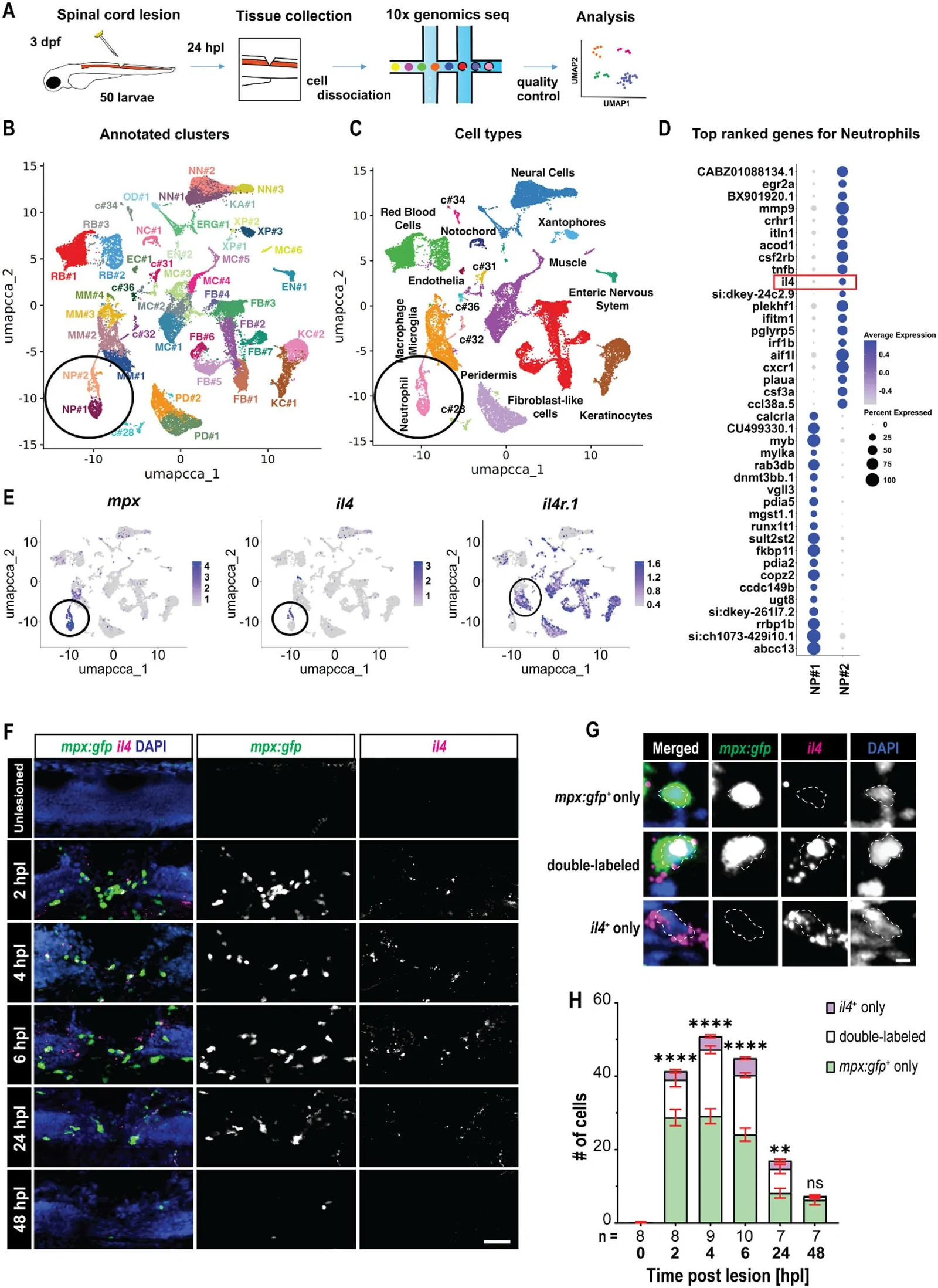
Il4 is mainly expressed by neutrophils at the spinal cord lesion site. **A**: A schematic of an experimental design for single-cell RNA sequencing (scRNA-seq) is shown. **B**, **C**: Cluster analysis of scRNA-seq data from the spinal lesion site at 24 hpl is shown (Docampo-Seara et al., 2024). Distinct clusters, including two neutrophil clusters (circled), are color-coded and annotated based on marker gene expression.** D**: *il4* ranks among the top 20 most highly expressed genes within the neutrophil sub-population NP#2. **E**: The dot plots identify neutrophils (*mpx*; circled in left and middle panels), show selective expression of *il4* in neutrophils and widespread expression of putative Il-4 receptor *il4r.1* (macrophage/microglia subpopulations are circled in right panel). **F**-**H**: A time-course analysis of il4 expression by HCR indicates expression of *il4* mainly in neutrophils (*mpx:gfp*^+^) during spinal cord regeneration (F). High-magnification images show examples of single and double-labelled cells (G). A quantification of *il4* expression is shown in H (Two-way ANOVA, interaction (F (10,128) = 24.35, p < 0.0001), time (F (5,128) = 109.3, *p *< 0.0001), and cell category (F (2,128) = 201.1,* p* < 0.0001). Tukey’s multiple comparisons test: in *mpx:gfp*^+^, *il4*^+^ double-labelled cells comparisons, 0 h vs. 2, 4 or 6 hpl (****p < 0.0001), 0 h vs. 24 hpl (**p = 0.0043), 0 h vs. 48 hpl (ns indicates no significance)). Error bars show SEM. Scale bar: 50 μm (F), 5 μm (G). Images are shown as maximum intensity projections of Z-stacks encompassing the spinal cord region (F, G: *n* = 5 slices, step size = 1 μm)

To determine potential signaling molecules that mediated the pro-regenerative effect of neutrophils on the lesion microenvironment, we interrogated previously generated scRNA-seq data sets on lesioned and unlesioned site tissue at 24 hpl [32]. This data set contained all major neural and non-neural tissues, including two clusters of neutrophils (*mpx*^+^) (Fig. 4A-C). To find selectively expressed signaling molecules in neutrophils, we performed a top-ranked gene expression analysis for these clusters and found high expression levels of *il4* in neutrophil population #2 (Fig. 4D). This expression was highly enriched in that neutrophil cluster compared to all major cell types. Interestingly, most other cell types, including macrophages/microglia, expressed *il4r.1*, a putative receptor for Il-4 (Fig. 4E), making the cytokine a good candidate to mediate neutrophil to macrophage communication.

To verify expression of *il4* in neutrophils, we performed in situ detection of *il4* mRNA in neutrophil reporter animals (*mpx:gfp*) in unlesioned animals at 3 dpf, and after injury at 2, 4, 6, 24, 48 hpl. Quantifications indicated at 2 hpl 10.25 *il4*-expressing neutrophils (*il4*^+^ / mpx:*gfp*^+^), which represented 81.1% of all *il4*-expressing cells; at 4 hpl, 18.11 neutrophils expressed *il4* (83.2*%* of all *il4*-expressing cells); *at* 6 hpl 16.20 neutrophils (77.9% of all *il4*-expressing cells); at 24 hpl 6.57 neutrophils (74.2% of all *il4*-expressing cells); and at 48 hpl 0.86 neutrophils (75% of all *il4*-expressing cells). Hence, the majority of *il4* expression occurred in neutrophils and was highest at 4 and 6 hpl. Conversely, at 2 hpl, 26.3%, at 4 hpl 38.4%, at 6 hpl 40.2%, at 24 hpl 44.7%, and at 48 hpl 12% of all neutrophils were *il4-positive.* The large population of neutrophils that was negative for an *il4* signal possibly corresponded to neutrophil population NP#1 seen in scRNA-seq not to express *il4* (Fig. 4F-H). However, it has to be noted that transcriptomics provide data on neutrophils at 24 hpl only, such that we cannot exclude that neutrophils that express *il4* during early phases after injury may have had an otherwise different gene expression profile from that at 24 hpl.

Ablation of neutrophils also reduced detectability of *il4* by qRT-PCR in unlesioned animals (by 28.7%) and showed a tendency to reduction at 4 hpl (by 21.6%), but not at 24 and 48 hpl, when neutrophil numbers were back to control levels (Suppl. Figure 9 A, B). This relatively modest reduction was likely due to the inclusion of *il4*-expressing cells in other tissues. For example, we detected cellular labelling of *il4* that did not coincide with the *mpx:gfp* transgene in the CHT (Suppl. Figure 10 A, B). Those uncharacterized cells appeared restricted to the CHT, but would have been inadvertently been included in our tissue sampling method. To address that issue, we used spatially more resolved HCR. Indeed, the *il4* mRNA HCR analysis following neutrophil ablation demonstrated an 80.46% reduction in the number of *il4*^*+*^ neutrophils at 6 hpl in the lesion. At 24 hpl, when overall neutrophil numbers were not different anymore from controls in our previous assays (cf. Fig. 1 A, B), there was still a very small reduction in the number of *il4*^*−*^ neutrophils (Suppl. Figure 9 C-G). Hence, *il4* was enriched in specific neutrophils in the lesion and neutrophil ablation reduced abundance of *il4* expression.

### Loss of il4 delays spinal cord regeneration

To directly test the importance of *il4* for spinal cord regeneration, we again generated somatic mutants. Gene disruption in somatic mutants efficiently mutated the targeted restriction enzyme recognition (Suppl. Figure 11 A). Moreover, mRNA expression was also strongly reduced, as indicated by qRT-PCR analysis (up to 75%; Suppl. Figure 11B). To further validate results, we used a previously published *il4* germline mutant [33].

Numbers of neutrophils and macrophages in the CHT, expression of major cytokines (*il1b*,* tnfa*,* tgfb1a*,* tgfb3*) and overall morphology were not affected in unlesioned *il4* somatic mutants (Suppl. Figure 11 C-J), indicating that lack of *il4* function did not lead to major aberrations of morphological development and development of the immune system.

**Fig. 5:**
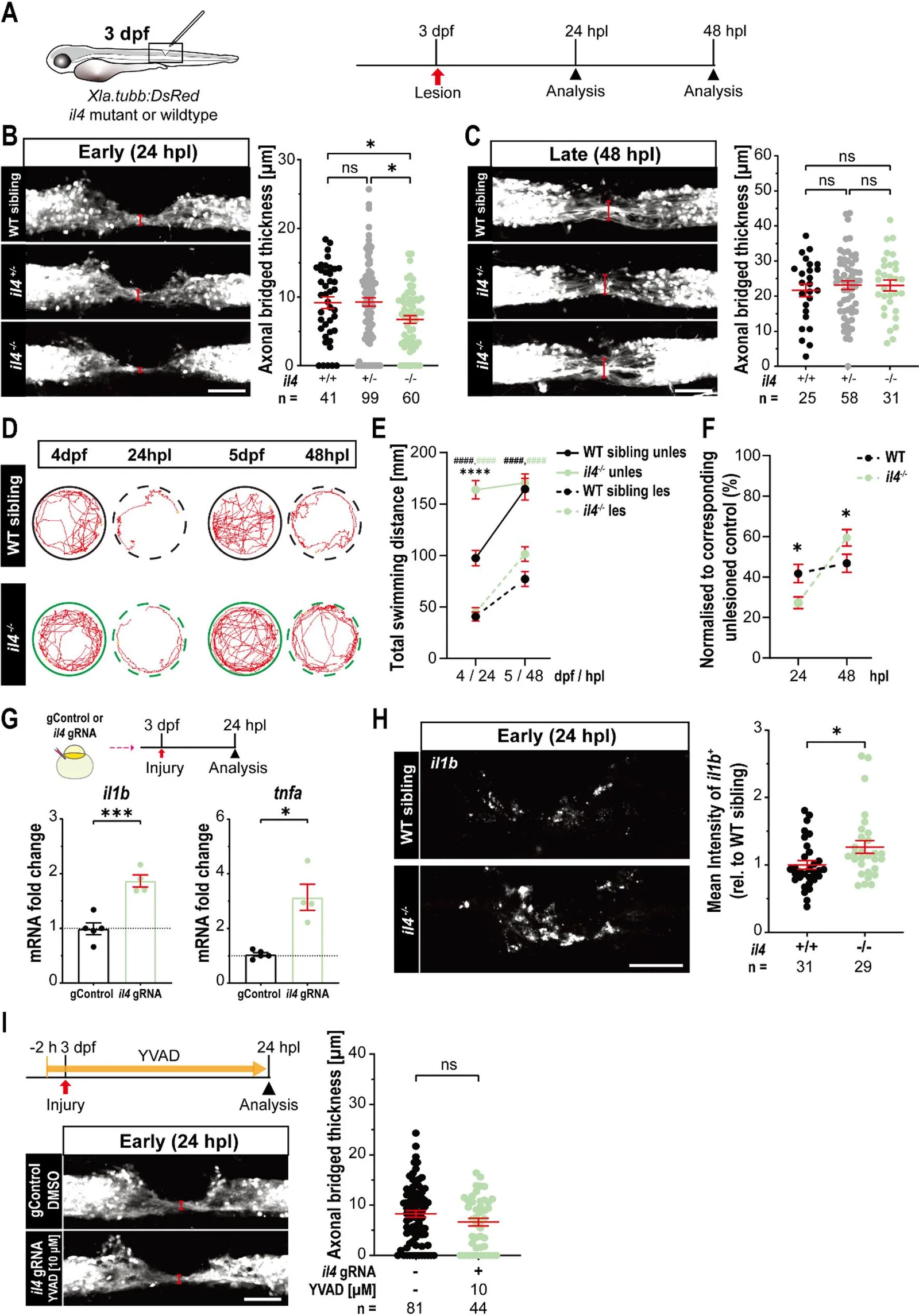
*il4* disruption delays spinal repair by increasing expression of *il1b*. **A**: A schematic timeline for assessing spinal cord regeneration in *il4* mutants with transgenically labelled neurons (*Xla.tubb:DsRed* fish) corresponding to (B-F, and I), is shown. **B**, **C**: In *il4* homozygous mutants (*il4*^-/-^), axonal bridge thickness is reduced, compared to wild type (WT) or heterozygote (*il4*^+/-^) siblings at 24 hpl (B), but not at 48 hpl (C). (B: Kruskal-Wallis test, *p* = 0.01. Dunn’s multiple comparisons test: *il4*^+/+^ vs. *il4*^-/-^ (*p = 0.0461), *il4*^+/-^ vs. *il4*^-/-^ (*p = 0.0154), ns indicates no significance. C: One-way ANOVA, *p* = 0.7822. ns indicates no significance). **D**, **E**: Unlesioned *il4*^-/-^ larvae exhibit greater locomotor activity compared to unlesioned WT siblings at 4 dpf. The lesion causes a significant reduction in swimming distance compared to unlesioned larvae (E). Representative swimming behavior tracks of WT and *il4*^-/-^ sibling larvae at different time points are shown (D). (WT sibling unlesioned n = 28, *il4*^-/-^ unlesioned n = 47, WT sibling lesioned n = 42, *n il4*^-/-^ lesioned n = 54. Two-way ANOVA, interaction (F (5,515) = 4.681, *p* = 0.0003), time (F (1,515) = 79.68, *p* < 0.0001), and treatment (F (5,515) = 83.58, p < 0.0001). Tukey’s multiple comparisons test: unlesioned vs. lesioned (####p < 0.0001), WT sibling unlesioned vs. *il4*^-/-^ unlesioned (****p < 0.0001)). **F**: Mutant larvae for *il4* show reduced recovery in swimming distance compared to wildtype at 24 hpl (normalized to respective unlesioned control; Two-way ANOVA using a mixed-effects model, interaction (F (1,28) = 15.83, p = 0.0004), time (F (1,53) = 0.0238, p = 0.8781), and genotype (F (1,53) = 30.29, *p* < 0.0001). Šídák's multiple comparisons test: *p = 0.0148 at 24 hpl, *p = 0.0270 at 48 hpl). **G**: A qRT-PCR analysis shows that *il1b* and *tnfa* mRNA expression are significantly upregulated in *il4* somatic mutants, compared to gControl-injected larvae. Each dot represents one biological replicate in each independent experiment, comprising 35 larvae. (Two-tailed unpaired t-test: *il1b* (***p = 0.00090), *tnfa* (*p = 0.0210)). H: The *il4*^-/-^ larvae increase the mean intensity of *il1b* fluorescence (HCR) at 24 hpl. (Two-tailed unpaired Mann–Whitney test: *p < 0.0194). I: 10 µM YVAD treatment rescues axonal bridge thickness in *il4* somatic mutant compared to wild type at 24 hpl. (Two-tailed unpaired Mann–Whitney test: ns indicates no significance). Error bars show SEM. Scale bar: 50 μm (B, C, H, I). Images are shown as maximum intensity projections of Z-stacks encompassing the spinal cord region (B, C, I: n = 35 slices, step size = 1 μm; H: *n* = 5 slices, step size = 1 μm)

To assess effectiveness of axonal regeneration in somatic *il4* mutants, we measured axonal bridge thickness as before. This revealed a 38.1% reduction at 24 hpl compared to control gRNA-injected larvae, with no significant difference observed anymore at 48 hpl (Suppl. Figure 12 A-D). Similarly, homozygous *il4* germline mutants showed a 26.6% reduced thickness of the axon bridge at 24 hpl, but no difference anymore at 48 hpl. Heterozygous siblings were unaffected (Fig. 5A-C). This indicated a delay in axon regrowth when *il4* was disrupted, similar to the effect of neutrophil ablation.

Next, we determined recovery of swimming function in *il4* mutants. We could not find differences to wildtype siblings at 24 and 48 hpl. However, we noticed that unlesioned *il4* mutants exhibited ~ 70% longer swim distances than their wildtype siblings, indicating a possible effect of Il-4 on motor system development. Studies in mammals have shown synaptic alterations and hyper-excitability following *Il-4* disruption, which may be related to our observation [34, 35] (Fig. 5D, E). Therefore, we normalized swim performance of lesioned larvae to that of their respective unlesioned siblings. We found a relatively worse recovery of *il4* mutants at 24 hpl, compared to wildtype animals. At 48 hpl, their relative swimming capacity was even better than that of wildtype animals (Fig. 5F). This data suggested potentially reduced recovery of axonal bridging and swimming behaviour in *il4* mutant animals at 24 hpl, similar to the effects of neutrophil ablation. However, because baseline swimming performance of the mutant strongly differs from that of wild type animals, the normalisation to the respective unlesioned genotype may have introduced bias to the analysis.

### Rescue of regeneration by reducing increased il1b levels in il4 mutants

To determine possible causes for the delayed regeneration when *il4* is disrupted, we characterized the immune response. First, we analyzed numbers of neutrophils and macrophages after spinal cord injury at 4, 24, and 48 hpl. We found no changes at early timepoints, but increased presence of neutrophils (78.6%) and macrophages (52.8%), at 48 hpl (Suppl. Figure 12E-H) in somatic *il4* mutants, indicating impaired resolution of inflammation after spinal cord injury when *il4* was disrupted. This may be related to the pro-resolving function of Il-4 that has previously been reported [36].

To find out whether lack of *il4* expression would increase the abundance of pro-inflammatory cytokines in the lesion, similar to what was observed after neutrophil ablation, we determined expression levels of *il1b* and *tnfa* in *il4* somatic mutants. Indeed, qRT-PCR indicated a doubling of *il1b* expression and three-fold more *tnfa* expression (Fig. 5G). In addition, using in situ detection of *il1b* mRNA, we found a 24.2% increase in labelling intensity in *il4* germline mutant animals compared wildtype siblings at 24 hpl (Fig. 5H).

Next, we tested whether inhibiting the elevated Il-1β release with YVAD could rescue axon regrowth in *il4* germline mutants. Indeed, the reduced thickness of the axonal bridge in lesioned mutants was rescued to that observed in wildtype animals by exposing the mutant larvae to 10 µM YVAD, as after neutrophil ablation (Fig. 5I). Hence, changes measured in axon regeneration and immune response in *il4* mutants were similar to those seen after neutrophil ablation and could also be reversed by YVAD treatment, which inhibits Il-1β processing.

### Il4 over-expression rescues lack of neutrophils

Based on the similarities between the phenotypes of neutrophil ablation and *il4* mutation, we hypothesized that Il-4 signaling may be the major mechanism by which neutrophils accelerate regeneration. To test this hypothesis, we aimed to rescue delayed regeneration in animals in which neutrophils were ablated by over-expressing *il4*. We did this by using a heat-shock inducible plasmid that was injected into the fertilized egg in a transient transgenesis approach. A single heat-shock at 2 h before injury led to a ~ 100-fold increase in *il4* mRNA detected by qRT-PCR in lesion site tissue of neutrophil-ablated animals at 24 hpl (Suppl. Figure 13 A, B). Using in situ detection of *il4* mRNA, we observed that the labelling intensity at 4 and 24 hpl increased by 12-fold and 2.5-fold respectively in animals injected with the *il4* overexpression plasmid, compared to the neutrophil ablation group without injection of the *il4* overexpression plasmid (Suppl. Figure 13 C-F). These observations indicated that after a single heat-shock the plasmid injection led to high levels of *il4* over-expression during the relevant experimental time period.

**Fig. 6:**
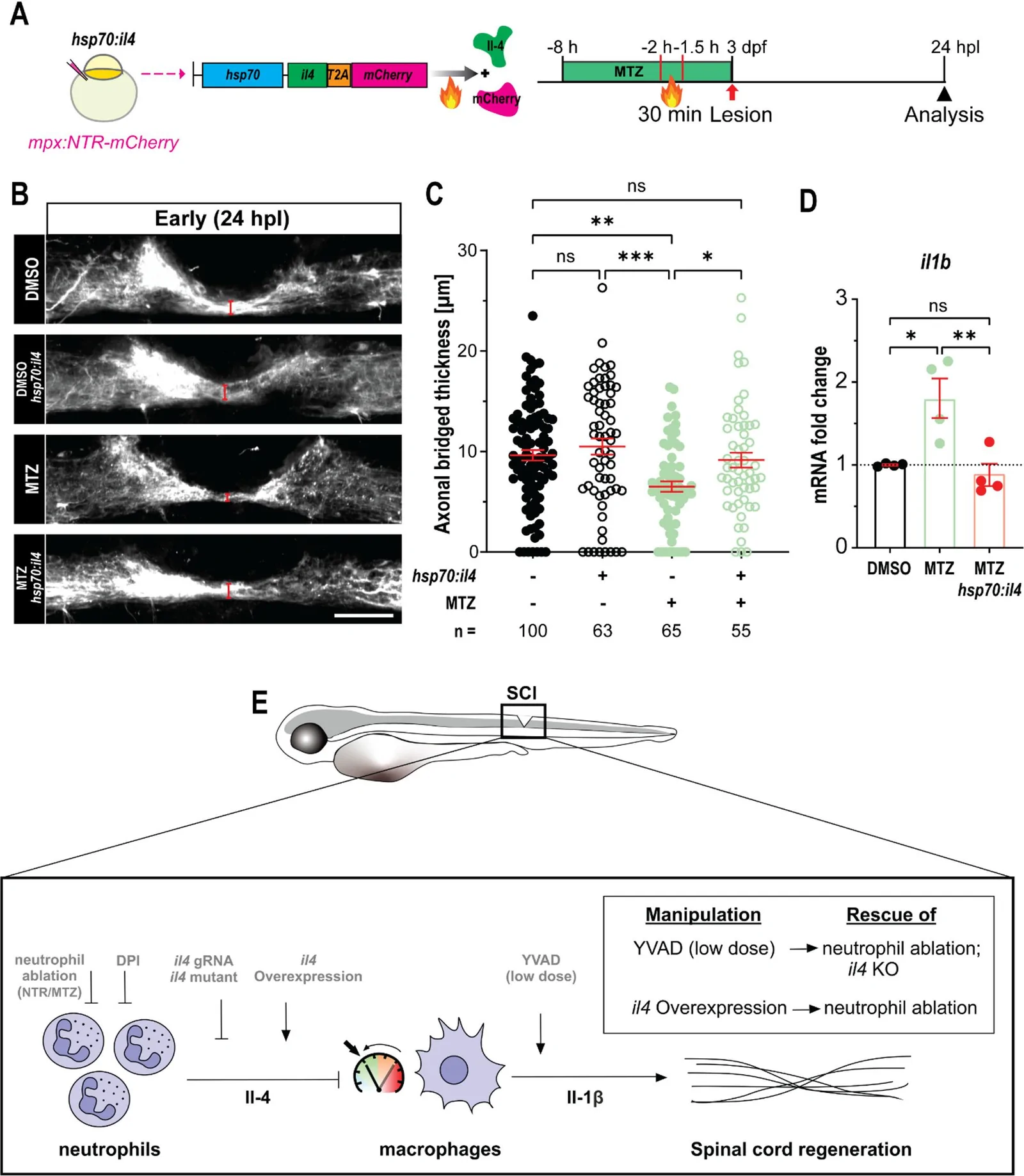
Overexpression of *il4* rescues spinal cord regeneration after neutrophil ablation. **A**: A schematic indicating the *hsp70:il4* plasmid construct and experimental timeline for (B-D) is shown. **B**, **C**: Heat-shock-induced *il4* overexpression increases the thickness of the regenerative axonal bridge in neutrophil-ablated larvae at 24 hpl, as revealed by anti-acetylated tubulin labelling, compared to neutrophil ablation alone, to that observed in non-heat shocked and non MTZ-treated controls. (Two-way ANOVA, interaction (F (1,279) = 1.851, *p* = 0.1748), hsp70:il4 (F (1,279) = 7.316, *p* = 0.0073), and MTZ treatment (F (1,279) = 11.88, *p *= 0.0007). Tukey’s multiple comparisons test: *p = 0.0363, **p = 0.0016, ***p = 0.0002, ns indicates no significance). **D**: A qRT-PCR analysis at 24 hpl shows that heat-shock-induced *il4* overexpression reduces *il1b* expression in neutrophil-ablated larvae to those in control larvae. (One-way ANOVA, *p* = 0.0050. Tukey’s multiple comparisons test: DMSO vs. MTZ (*p = 0.0143), DMSO vs. MTZ hsp70:il4 (**p = 0.0064), ns indicates no significance). **E**: As schematic of the proposed promotion of regeneration by neutrophils via control of macrophage il1b expression is shown. Experimental manipulations are depicted in grey and the table indicates rescue experiments. Error bars show SEM. Scale bar: 50 μm (B). Images are shown as maximum intensity projections of Z-stacks encompassing the spinal cord region (B:* n* = 35 slices, step size = 1 μm)

Next, we measured axonal bridge thickness as a measure of regenerative success in larvae in which we combined neutrophil ablation with *il4* over-expression. Over-expression of *il4* alone after spinal lesion did not change the axonal bridge thickness compared to non-heat shocked and non-MTZ treated controls. Ablation of neutrophils with MTZ treatment alone, reproduced the previously described reduction in axonal bridge thickness at 24 hpl (cf. Fig. 1E). The combination of neutrophil ablation and heat-shock induced over-expression of *il4* increased the thickness of the axonal bridge to values that were not distinguishable anymore from those in controls (Fig. 6A-C). This demonstrated a full phenotypic rescue of reduced axon regrowth caused by neutrophil ablation by over-expression of *il4.*

To determine whether *il4* overexpression would also rescue the increased expression of pro-inflammatory cytokines after neutrophil ablation, we performed qRT-PCR measurements of *il1b* mRNA in the lesion. Neutrophil ablation led to an 80.6% increase in expression levels of *il1b*, compared to vehicle-treated controls at 24 hpl, which was comparable to our earlier experiment (cf. Fig. 2 C). Over-expression of *il4* in neutrophil-ablated larvae, reduced the expression level of *il1b* to that seen in vehicle-treated, non-heat-shocked controls (Fig. 6D). Interestingly, successful over-expression of *il4* in lesioned animals without ablation of neutrophils had no measurable effect on *il1b* expression (Suppl. Figure 13G), suggesting that endogenous levels of *il4* could already have had maximal effects on *il1b* expression and that additional mechanisms contributed to regulation of *il1b* expression. Hence major changes in regenerative axon growth and in the cytokine milieu were rescued by over-expression of *il4* alone. Together, this suggested an important role for neutrophil-derived Il-4 in in accelerating axonal regeneration by regulating *il1b* expression in macrophages (Fig. 6E). In addition, neutrophils also expressed *il1b* [22] and it is possible that Il-4 also regulates *il1b* expression in neutrophils or other cell types.

## Discussion

Here we identify a pro-regenerative sub-population of neutrophils in zebrafish. Using a series of rescue experiments, we propose that these neutrophils accelerate axon regrowth and recovery of swimming behavior mainly via Il-4 signaling, which in turn controls *il1b* expression in macrophages and potentially other cells.

Our data suggest that neutrophil-derived Il-4 induces a pro-regenerative phenotype in macrophages. In mouse spinal cord injury, it has been shown that initial levels of IL-4 after spinal cord injury are very low, suggesting that an *il4*-expressing subpopulation of neutrophils is very small, if it exists. Importantly, exogenous supply of IL-4 leads to improved functional recovery, reduced tissue damage and increased expression of tissue repair markers by macrophages [37, 38]. Similar observations have been made in a myocardial infarction model, where IL-4 stimulated anti-inflammatory phenotypes in both macrophages and neutrophils [36]. In a zebrafish, in a model of hair cell regeneration in the lateral line nerve, macrophage-derived Il-4 promotes synaptogenesis [39]. Il-4 also promotes neurogenesis in a neurodegeneration model in the adult zebrafish brain [40]. These examples indicate direct and indirect pro-regenerative roles of Il-4.

In mammals, neutrophils rarely secrete IL-4 [41, 42]. However, in a mouse model of neonatal encephalopathy, caused by hypoxia-ischemia, a late-arriving subpopulation of neutrophils has recently been discovered that promotes repair and expresses *Il-4*, but the functional role of the cytokine has not been tested in that model [43].

Il-4 from neutrophils could act on macrophages via the Il4r.1 receptor, which is expressed by macrophages, but also via other regeneration-relevant cell types, such as *il4r.1* expressing fibroblasts [44]. The potentially complex down-stream actions of Il-4 in zebrafish warrant further investigations.

Neutrophils may also have other positive functions for regeneration. For example, in mammals, some neutrophils secrete growth factors, such as NGF, IGF-1 or Oncomodulin, which promote recovery after optic nerve or spinal cord lesion [17, 45]. Neutrophils can also form extracellular material in a regeneration context that may aid regeneration [46]. Nevertheless, in the larval zebrafish injury model, exogenous Il-4 was sufficient to replace the presence of neutrophils in the injury site for axon regrowth, indicating that Il-4 plays a decisive role in neutrophil signalling.

Il-1β is a major determinant of regenerative success. Perturbations of the immune response after injury, e.g., in macrophage-less *irf8* mutants, lead to increased levels of *il1b* expression, which is detrimental to regenerative success and can be rescued by interfering with Il-1β processing directly, or indirectly by treatment with anti-inflammatory drugs, both of which rescue regeneration [22, 23, 30]. In mammals, IL-1β receptor antagonist treatment leads to reduced inflammation and neutrophil recruitment to a spinal cord injury site [47]. However, levels and timing of Il-1β presence in the injury site are critical for regenerative success. Here we show that neutrophils control expression of *il1b* in macrophages and that reducing Il-1β processing to the right dose is critical for rescue experiments. Previously, we observed that reducing Il-1β processing during early, but not late phases of regeneration in wildtype animals impaired axon regrowth, such that early expression of *il1b*, likely from neutrophils themselves, promotes regeneration [22].

Lack of neutrophils also leads to increased expression of *tnfa*, which promotes axon regrowth and regenerative neurogenesis on its own [22, 27]. Apparently, the positive functions of *tnfa* are outweighed by the negative ones of *il1b* for regeneration. There are also complex feedback interactions between these two cytokines, as *tnfa* is under positive control by *il1b* and *il1b* is under negative control by *tnfa* in the lesion site [22]. In this way YVAD could also act in additional cytokines that may be in part responsible for the observed rescue of regeneration.

A potential technical limitation of our study is that macrophages, which are highly phagocytotic after spinal cord injury, also phagocytose nitroreductase-ablated neutrophils, leading to a higher proportion of neutrophil-loaded macrophages than in controls. Neutrophil phagocytosis by macrophages can lead to altered reactions to IL-4 [48]. This potential confounder is mitigated by our observation that preventing neutrophil infiltration with DPI led to similar regeneration phenotypes. Moreover, when only *il4* is disrupted without killing the neutrophils, the resulting regeneration and cytokine milieu changes are comparable to those observed after neutrophil ablation. In particular, in both situations *il1b* expression is increased and axonal phenotypes are fully rescued by reducing Il-1β processing. We propose that there are conserved aspects in the innate immune response to spinal cord injury between larval zebrafish and mammals in that IL-4 reprograms macrophages to a pro-repair phenotype. A notable difference is that IL-4-expressing neutrophils are rare in the initial immune response in mammals, but not in zebrafish. Therefore, this novel sub-population of *il4-*expressing neutrophils in larval zebrafish could partially explain the remarkable regenerative success in that species. Our findings also support the notion that therapeutic efforts in mammals may be directed to inducing more pro-regenerative IL-4-producing neutrophils.

## Materials and methods

### Animals

All zebrafish lines were raised and kept under standard conditions [49, 50]. Zebrafish experiments were performed under state of Saxony licenses TVV 36/2021, TVV 45/2018, and holding licenses DD24-5131/364/11, DD24-5131/364/12. For experimental conditions, larvae up to an age of 5 days post-fertilization (dpf) were used of the following lines: wild type (AB); *Tg(mpx:GFP)*^*i114*^, abbreviated as *mpx:gfp* [51]; *TgBAC(mpx:GAL4-VP16)*^*sh267*^, *Tg(UAS-E1B:NTR-mCherry)*^*c264*^, abbreviated as *mpx:NTR-mCherry* [52, 53]; *Tg(mnx1:GFP)*^*ml2*^, abbreviated as *mnx1:gfp* [54]; *Tg(mpeg1:mCherry)*^*gl23*^, based on the regulatory sequences of the *mpeg1.1* gene and abbreviated as *mpeg1:mCherry* [55]; *Tg(Xla.Tubb:DsRed)*^*zf148*^, abbreviated as *Xla.Tubb:DsRed* [56]; *il4*^*umc18/umc18*^, abbreviated as *il4* mutants [33]. For all experiments, zebrafish larvae were kept in E3 medium [49] without methylene blue supplement.

### Image acquisition, processing, and presentation

Images were acquired using the systems described in each subsection. Images were processed using ImageJ (http://rsb.info.nih.gov/ij/; version 2.3.0/1.54), and Adobe Photoshop 2025 (version 25.6), and Figure plates were prepared using Adobe Illustrator 2024 (version 28.7.5). Images of the whole animal, zebrafish trunk or lesion site are lateral views (dorsal up; rostral left), if not indicated differently.

### Spinal cord lesion

Lesions of larvae were performed as previously described [28]. Briefly, at 3 dpf, zebrafish larvae were anaesthetized in E3 medium [57] containing 0,02% of MS-222 (Sigma-Aldrich, St. Louis, MO, USA; Cat#A5040). Larvae were then transferred to an agarose plate. After removal of excess water, larvae were placed in lateral positions. A transection of the entire spinal cord was made using a 30G x ½″ hypodermic needle (Becton Dickinson, Franklin Lakes, NJ, USA; Cat#613–3942) at the level of the urogenital pore, without injuring the notochord. After surgery, larvae were returned to E3 medium for recovery and kept at 28.5 °C. Larvae in which the notochord had been inadvertently damaged were excluded from further analysis.

### CRISPR/Cas9 manipulation

CRISPR/Cas9-mediated mutagenesis was achieved following a previously published protocol [30]. CRISPR gRNAs were selected using the CRISPRscan (www.crisprscan.org/gene) web tool [58], and the CRISPOR (http://crispor.org) web tool [59]. gRNAs were chosen based on the CRISPRScan score (preferentially > 60) and the CRISPOR scores (high specificity, high efficiency and low out-of-frame scores), and on the availability of a suitable restriction enzyme recognition site (www.snapgene.com) that would be destroyed by the gRNA. Additional criteria were to avoid the first exon and to target functional domains. All gRNAs and tracrRNA were ordered from Merck KGaA (Darmstadt, Germany). 1 nL of gRNA mix (1 µL of Tracer (5nM, Merck KGaA, Darmstadt, Germany; Cat#TRACRRNA05N), 1 µL of each gRNA (20µM, Merck KGaA, Darmstadt, Germany), 1 µL of phenol red (Sigma-Aldrich, St. Louis, MO, USA; Cat#P0290) and 1 µL of Cas9 (20µM, New England Biolabs, Ipswich, MA, USA; Cat#M0386M) was injected into one-cell stage embryos. gRNA efficiency was tested by restriction fragment length polymorphism analysis (RFLP) in a proportion of larvae for each experiment as an internal experimental control [30]. Sequences for gRNAs, primers and the restriction enzymes (RE, New England Biolabs, Ipswich, MA, USA) used for RFLPs are provided in Supplementary Table 1. The *il4* gene (ENSDARG00000087909) was targeted by injection of gRNA for exon 4 (5’- CTTCATTGTGCATTCCCCCG − 3’). The *il1b* gene (ENSDARG00000098700) was targeted by co-injection of gRNAs for exon 3 (5’- CTGTTCAGATCCGCTTGCAA − 3’) and exon 5 (5’- GCTTCCGGCTCTCAGTGTGA − 3’). All experiments conducted with somatic mutants were carried out with a control injected group with a non-targeting gRNA (5’- UUACCUCAGUUACAAUUUAU − 3’).

### Generation of heat shock plasmid and treatment

To create the *pTol-hsp70l:il4-T2a-mCherry* plasmid, we modified the *pTol-hsp70I:sema4ab-T2a:mCherry* vector [32]. We PCR-amplified the *il4* coding sequence from genomic cDNA with specific primers (Supplementary Table 2). Then, we performed a simultaneous multiple fragment ligation in the opened backbone vector. The final construct *pTol-hsp70l:il4-T2a:mCherry* was verified by sequencing, qRT-PCR for over-expression and *il4* mRNA fluorescence (HCR-FISH) after injection.

### In situ hybridization chain reaction (HCR-FISH)

Hybridization chain reaction (HCR) RNA fluorescence in situ hybridization (Molecular Instruments, Los Angeles, CA, USA) was performed according to the manufacturer’s instructions with minor modifications [60, 61]. In brief, terminally anesthetized larvae were fixed overnight in 4% paraformaldehyde (PFA, Thermo Scientific, Waltham, MA, USA; Cat#28908) in phosphate buffered saline (PBS, Mediakitchen of TUD, Dresden, Germany) at 4 °C and then transferred to 100% Methanol (MeOH, Carl Roth, Karlsruhe, Germany; Cat#4627.1) at -20 °C overnight for permeabilization. The next day, larvae were rehydrated in a graded series of MeOH at room temperature (RT) and subsequent washes in phosphate buffered saline with 0.1% Tween (PBST). The larvae were treated with 100% acetone (pre-chilled to -20 °C. Carl Roth, Karlsruhe, Germany; Cat#7328.1) at -20 °C for 10 min, and washed with PBST (3 × 5 min). Thereafter, larvae were treated with 1 ml proteinase K (Roche Diagnostics GmbH, Mannheim, Germany; Cat#3115828001) at a concentration of 12.5 µg/mL for 60 min at RT, followed by post-fixation in 4% PFA for 15 min RT and washes with PBST (3 × 5 min). Pre-hybridisation was performed using 500 µL of pre-warmed probe hybridisation buffer (Molecular Instruments, Los Angeles, CA, USA) for 30 min at 37 °C [60]. The probe sets, which were designed based on the NCBI sequence by Molecular Instruments [60], were prepared using 2 pmol of each probe set in 500 µL of pre-warmed hybridisation buffer. Then, buffer was replaced with probe sets and incubated for 16 h at 37 °C. On the following day, samples were washed 4 × 15 min with pre-warmed probe washing buffer (Molecular Instruments, Los Angeles, CA, USA) at 37 °C followed by 2 × 5 min in 5x sodium chloride sodium citrate with 0.1% Tween (SSCT) at RT. Larvae were then treated with 500 µL of room temperature equilibrated amplification buffer (Molecular Instruments, Los Angeles, CA, USA) for 30 min at RT. Hairpin RNA preparation was performed following the manufacturer’s instructions. Samples were incubated with Hairpin RNAs for 16 h in the dark at RT. Finally, samples were washed for 2 × 45 min in 5 x SSCT with DAPI (1:1000, Sigma-Aldrich, St. Louis, MO, USA; Cat#D9564), followed by 3 × 5 min in 5 x SSCT at RT. Samples were mounted in 75% glycerol (Carl Roth, Karlsruhe, Germany; Cat#3783.1) in PBS and imaged using a Dragonfly spinning disc microscope (Andor/Oxford Instruments, Belfast, UK).

### Combining EdU with motor neuron labelling

Labelling newly generated motor neurons followed an established protocol [28]. All incubations were performed at room temperature unless stated otherwise. Briefly, lesioned and unlesioned larvae were incubated in E3 medium containing 1% dimethylsulfoxide (DMSO, Sigma-Aldrich, St. Louis, MO, USA; Cat#D8418) and 1:100 EdU (Click-iT™ Plus EdU Cell Proliferation Kit. Thermo Fisher Scientific, Waltham, MA, USA; Cat#C10640) for the period determined by the experimental design. Terminally anesthetized larvae were fixed in 4% PFA overnight at 4 °C and permeabilised in 100% MeOH at -20 °C for at least overnight. After removing the head with micro scissors, larvae were then rehydrated, and incubated in proteinase K and re-fixed in 4% PFA. Then, larvae were incubated with the Click-iT™ Plus EdU Cell Proliferation Kit (Alexa Fluor™ 647 dye) as described by the manufacturer. Subsequently, samples were washed in PBST and incubated with blocking buffer solution (2% BSA, 1% DMSO, 1% Triton, 10% Normal donkey serum, in PBST) for 1 h and then with a chicken anti-GFP antibody, (1:200, Abcam, Cambridge, UK; Cat# Ab13970) for 72 h at 4 °C. Secondary antibody (Alexa Fluor^®^ 488 Donkey Anti-Chicken IgY [IgG], 1:200, Jackson ImmunoResearch Laboratories, West Grove, PA, USA; Cat#703-545-155) incubation took place overnight at 4 °C. Finally, samples were washed in PBST and transferred to 70% glycerol in PBST, mounted and imaged. Imaging was performed using a Dragonfly spinning disc microscope. Images were analysed using ImageJ [62, 63]. Newly generated motor neurons were counted by assessing the number of double-positive cells for GFP and EdU. Cells were counted manually along the Z-stack, ensuring counting individual cells in single optical sections, in 50 μm windows located at both sides of the injury site (100 μm in total). To mitigate bias, experimental conditions were occluded for the analysis. At least two independent experiments were performed. Individual experiments were merged and analysed for statistical significance.

### Fluorescent immunohistochemistry

Anti-Mfap4 and anti-Mpx immunohistochemistry on whole-mount zebrafish larvae was carried out as an established protocols [64]. Briefly, terminally anesthetized larvae were fixed in 4% PFA overnight at 4 °C. After removing the head with micro-scissors, larvae were permeabilized by subsequent incubation in acetone and proteinase K. Samples were re-fixed in 4% PFA, blocked in blocking buffer solution (2% BSA, 1% DMSO, 1% Triton, 10% Normal donkey serum, in PBST), and incubated over three nights with rabbit polyclonal anti-Mfap4 antibody (GeneTex, Irvine, CA, USA; Cat#GTX132692) or or rabbit polyclonal anti-Mpx antibody (GeneTex, Irvine, CA, USA; Cat# GTX128379) at a dilution of 1:200.

For anti-acetylated tubulin immunolabelling, terminally anesthetized larvae were fixed in 4% PFA for 1 h at room temperature. After removing the head with micro scissors, larvae were permeabilized by subsequent incubation in acetone and proteinase K. Samples were re-fixed in 4% PFA, blocked with blocking buffer solution, and incubated over three nights with mouse monoclonal anti-acetylated tubulin antibody (Sigma-Aldrich, St. Louis, MO, USA; Cat#T6793) at a dilution of 1:300.

The corresponding secondary antibodies conjugated to Alexa Fluor dyes were obtained from Jackson ImmunoResearch (Cat#711-605-152, 715-605-151) and used at a dilution of 1:300 and incubated overnight at 4 °C. Finally, samples were mounted with 70% glycerol in PBST, and imaged using a Dragonfly spinning disc microscope.

### Measurement of axonal regrowth

As an indicator for successful axon regrowth, we determined the extent of continuous labelling of axons across the spinal cord injury site, termed axonal bridging, as previously described [22, 26]. We determined the thickness of the axonal bridge at its narrowest point. Spinal lesion was performed at 3 dpf as described in larvae above. Terminally anesthetized larvae were fixed in 4% PFA and immuno-labelled against acetylated tubulin (if axons were not transgenically labelled). Images were taken with a Dragonfly Spinning Disk microscope with a step size of 1 μm.

The bridge thickness was determined using a custom script in Fiji [62, 63]. Measurements were performed at the narrowest point of the axonal bridge, which was manually identified on a maximum intensity projection of the z-stack after binary segmentation. Data obtained from at least three independent experiments were quantified.

### Analysis of swimming behaviour

The swimming behaviour was assessed using the ZebraBox setup (ViewPoint Behavior Technology, Lyon, France). A spinal cord lesion was inflicted at 3 dpf, and its completeness was confirmed at 2 hpl in each larva. Subsequently, the larvae were individually arranged in 24-well plates, ensuring representation of all groups on each plate, and kept in an incubator. Larval movements were automatically tracked. Measurements encompassed three repetitions of the same protocol. The entire experimental procedure lasted 2 min. The first minute served as a rest period to allow the animals to adapt to the device environment. The second minute consisted of a 1-second vibration stimulus (frequency set to 1000 Hz; 100% target power), followed by 59 s without stimulation. For analysis, we focused on the full second-minute interval, which included the 1-second stimulus and the subsequent 59-second no-stimulus phase. The analysis was performed with ZebraZoom (https://zebrazoom.org/) [65], an open-source tracking software, to track the swimming paths as well as measure the swim distance of each larva. Two independent experiments were performed and the results were merged for statistical analysis.

### Quantitative RT-PCR

RNA was extracted using the Direct-zol RNA Microprep Kit (Zymo Research, Irvine, CA, USA; Cat#R2060) according to the manufacturer’s instructions (40 larvae/condition). The lesioned site or the corresponding unlesioned tissue was enriched by cutting away rostral and caudal parts from the larvae at positions approximately 3 somites away from the lesion site in the rostral and caudal direction. cDNA was synthesized using the Transcriptor High Fidelity cDNA Synthesis Kit (Sigma-Aldrich, St. Louis, MO, USA; Cat#5091284001) according to the manufacturer’s instructions. qRT-PCR was performed using the FastStart Universal SYBR Green Master (Sigma-Aldrich, St. Louis, MO, USA; Cat#4913914001) according to the manufacturer’s instructions and run in triplicates on a LightCycler 480SW instrument (Roche Diagnostics GmbH, Mannheim, Germany). At least three individual experiments were run and the data was analyzed using the LightCycler 96/384 application software v1.1 (Roche Diagnostics GmbH, Mannheim, Germany). Each condition was normalized to housekeeping gene (*β-actin*) and the experimental group was compared to controls by normalizing each value to the control average value. Primers were designed to span an exon–exon junction using Primer-BLAST software or obtained from the literature. Primer sequences are given in Supplementary Table 3. 

### Immune cell counting in whole-mounted larvae

Larvae were lesioned at 3 dpf and fixed for 1 h in 4% PFA at different time points. Larvae were washed in PBST and transferred to glycerol (70% in PBST), mounted and imaged immediately using a Dragonfly Spinning Disk microscope. Images were analysed using Fiji with the experimental condition unknown to the observers. The number of cells in the vicinity of the injury site was counted in a volume of interested that was centered on the lesion site (width, X-axis − 200 μm, height, Y-axis − 75 μm, depth, Z-axis − 50 μm), right above the notochord. Cells were counted manually inside the window along the Z-stack with the experimental condition occluded. At least two individual experiments were carried out and merged.

### Acridine Orange labelling

Larvae of the *mpx:NTR-mCherry* line were incubated in 1 µg/ml Acridine Orange (Sigma-Aldrich, St. Louis, MO, USA; Cat#A6014) in E3 medium for 3.5 h prior to imaging [66]. Following incubation, fish were washed three times for 10 min each in E3. Larvae were then mounted in 0.5% low-melting-point agarose (Carl Roth, Karlsruhe, Germany; Cat#6351.1) in E3 for live imaging using a Dragonfly spinning disk confocal microscope. To quantify cellular debris, an observation window (width, X-axis = 585 μm; height, Y-axis = 100 μm; depth, Z-axis = 50 μm) was defined over the region of interest centred on the caudal hematopoietic tissue. The number of cells co-expressing mCherry and Acridine Orange signals was counted within this volume.

### Fluorescence intensity measurements

Images were analysed with Fiji ImageJ (https://imagej.net/ij/). The region of interest was defined as a rectangle of 250 × 100 μm, centred on the injury site and located right above the notochord. 50 Z-stacks were collapsed in sum intensity projections and then background subtraction was performed before analysing the mean intensity with a custom Fiji macro to automatize the process ([62]; [63]). Mean intensity was represented by normalizing each value to the control average value.

### Drug treatment and heat shock

Drug treatments and heat shock were performed according to the schematic timelines shown with the experiment. Larvae were incubated in E3 medium containing the drugs, with a final DMSO concentration of 1%. To inhibit caspase-1 activation of Il-1β, Ac-YVAD-cmk (Sigma-Aldrich, St. Louis, MO, USA; Cat# SML0429) [22] was dissolved in DMSO to a stock concentration of 10mM and used at a final concentration of 10, 25, and 50 µM. To block neutrophil infiltration by inhibiting NADPH oxidase, diphenyleneiodonium chloride (DPI, Sigma-Aldrich, St. Louis, MO, USA; Cat#D2926) [29] was dissolved in DMSO to a stock concentration of 10 mM and used at a final concentration of 100 µM. For targeted cell ablation of *mpx*^*+*^ cells, the Metronidazole (MTZ; MedChemExpress, Monmouth Junction, NJ, USA; Cat#HY-B0318) was dissolved in DMSO and used at a final concentration of 5 mM. For heat shock, larvae were kept in 50-mL conical centrifuge tubes filled with pre-warm E3 medium, which floated in a programmable thermostat-controlled water bath (VWR International BVBA, Leuven, Belgium). Heat shock was performed for 30 min at 38 °C, after which larvae were returned to 28.5 °C.

### scRNAseq analysis

A previously obtained Seurat object (NCBI GEO accession no. GSE276092) on lesion enriched tissue (24 hpl) was used [32]. Neutrophils were identified by enriched expression of *mpx* and subset for further analysis. For the differential gene analysis the FindAllMarkers function from Seurat (version 5.0.2) was used with default parameters. The “Wilcoxon rank sum test”, and p_val_adj (p-value adjusted) < 0.1 and avg_log2FC > = 0.25 (average log2 fold change) were used for selecting significantly expressed genes. Genes were ordered based on their avg_log2FC in descending order for each neutrophil cluster. The 20 top-ranked genes were then selected based on their representative expression of each cluster vs. the other (pct.1 > 0.2, pct.2 < 0.2).

A previously obtained Seurat object (ArrayExpress: E-MTAB-10379) on FACS-enriched immune cells in unlesioned and lesioned animals (24 hpl) was also used [27].

### Bias mitigation and statistical analysis

For all quantifications, investigators were blinded to the experimental conditions. Unless indicated, no data were excluded from analysis. The experiment was independently repeated at least twice, and a two-way ANOVA was used to evaluate treatment effects and to confirm consistency across experiments for data pooling [67]. All statistical analyses were performed using GraphPad Prism 10 (GraphPad Software Inc., version 10.6.0). All quantitative data were tested for normal distribution using the Shapiro-Wilk test. Parametric and non-parametric tests were used as appropriate. We used two-tailed Welch’s t-test, two-tailed Mann–Whitney test, Kruskal–Wallis test with Dunn’s multiple comparisons test, One-way ANOVA with Tukey’s multiple comparisons test, Two-way ANOVA with Tukey’s multiple comparisons test or Šídák’s multiple comparisons test. A one-tailed Mann-Whitney test was used for the comparison shown in Fig. 3G, based on a predefined directional hypothesis. Differences were considered statistically significant at *p* < 0.05. Unless otherwise stated, data are presented as mean ± standard error of the mean (SEM). Graphs were generated using GraphPad Prism 10. Unless otherwise indicated, each data point represents one animal.

## Supplementary Information

Supplementary Material 1.

Supplementary Material 2.

## Data Availability

All data and materials generated in this study are reported in the manuscript and are available from the corresponding author upon request.
