# Supplementary material for: A reparative neutrophil subpopulation accelerates spinal cord regeneration in zebrafish by controlling macrophage inflammation via Il-4
Source: J Neuroinflammation. 2026 May 26;23:308. doi: 10.1186/s12974-026-03878-0 (PMC13560386; doi:10.1186/s12974-026-03878-0)
Supplement: Supplementary file 1 [file 12974_2026_3878_MOESM1_ESM.docx]

**RFLP full uncropped Gels and Blots images.**

**A:** RFLP analysis shows disruption of genomic DNA after injection of *il1b* gRNAs targeting exon 3 and 5, respectively. This image corresponds to Suppl. Fig. 7B. **B:** RFLP analysis shows effective disruption of genomic DNA after *il4* gRNA injection by resistance to restriction digestion site of the targeted region (red arrows). This image corresponds to Suppl. Fig. 11A.
