# Supplementary material for: A reparative neutrophil subpopulation accelerates spinal cord regeneration in zebrafish by controlling macrophage inflammation via Il-4
Source: J Neuroinflammation. 2026 May 26;23:308. doi: 10.1186/s12974-026-03878-0 (PMC13560386; doi:10.1186/s12974-026-03878-0)
Supplement: Supplementary file 2 [file 12974_2026_3878_MOESM2_ESM.docx]

SUPPLEMENTARY FIGURES

**Suppl. Fig. 1 MTZ treatment efficiently ablates neutrophils.**

**A:** A schematic timeline of MTZ treatment and Acridine Orange (AO) staining for (B, C) is shown. **B, C:** Approximately 89.7% of neutrophils (magenta) are acridine orange positive (green) after MTZ treatment in the caudal hematopoietic tissue (CHT) (Two-way ANOVA, interaction (*F* (1,32) = 195.0, *p* < 0.0001), treatment (*F* (1,32) = 0.2442, *p* = 0.6246), and cell category (*F* (1,32) = 0.4931, *p* = 0.4876). Tukey’s multiple comparisons test: *****p* < 0.0001). **D:** A schematic timeline of anti-Mfap4 staining in unlesioned larvae for (E-F) is shown. **E, E’:** The *mpx:NTR-mCherry⁺* neutrophils (magenta) and Mfap4⁺ macrophages (green) in the CHT of DMSO- and MTZ-treated larvae. Arrowheads mark Mfap4⁺ macrophages engulfing neutrophil debris. **F:** The numbers of Mfap4⁺ macrophages are not significantly different between DMSO- and MTZ-treated groups in the CHT area. (Two-tailed unpaired t-test, ns indicates no significance). **G:** A schematic timeline of anti-Mfap4 staining in lesioned larvae for (H-I) is shown. **H, I:** MTZ treatment increases the number of *mpx:NTR-mCherry⁺*/ Mfap4⁺ cells at 4 hpl in the lesioned area. (Two-way ANOVA using a mixed-effects model, interaction (*F* (1.827,36.54) = 102.7, *p* < 0.0001), treatment (*F* (1,20) = 102.9, *p* < 0.0001), and cell category (*F* (1.827,36.54) = 30.51, *p* < 0.0001). Tukey’s multiple comparisons test: *****p* < 0.0001). **J, K:** MTZ treatment of *mpx:NTR-mCherry* transgenic animals significantly reduced the number of anti-Mpx^+^ neutrophils at 4 hpl in the lesioned area. (Two-tailed unpaired t-test: *****p* < 0.0001). Error bars show SEM. Scale bars, 100 μm (B, E), 50 μm (H,J) and 5 μm (E’, H’). Images are shown as maximum intensity projections of Z-stacks encompassing the CHT or spinal cord region (B, E, J : n = 35 slices, step size = 1 μm; H: n = 5 slices, step size = 1 μm).

**Suppl. Fig. 2** **Neutrophil ablation delays regenerative neurogenesis.**

**A:** A schematic timeline of assessing motor neuron proliferation for (B-E) is shown. **B, C:** Neutrophil ablation reduces the number of newly generated motor neurons (*mnx1:gfp⁺*/ EdU⁺) at 24 hpl, compared to the indicated different control conditions. MTZ treatment doesn’t affect the developmental neurogenesis of motor neuron at 4 dpf. Dashed squares indicate the region of quantification; arrowheads indicate double-labeled cells. High-magnification images of a double-labeled neuron (*mnx1:gfp⁺*/ EdU⁺) (B’), (Kruskal-Wallis test in unlesioned, *p* = 0.7817. Kruskal-Wallis test at 24 hpl, *p* = 0.0034, Dunn’s multiple comparisons test: **p* = 0.0281, ns indicates no significance). **D, E:** MTZ treatment does not affect the motor neuron neurogenesis (*mnx1:gfp⁺*/ EdU⁺) at 5 dpf and 48 hpl. (One-way ANOVA in unlesioned, *p* = 0.7109. One-way ANOVA at 48 hpl, *p* = 0.5492, ns indicates no significance). Error bars show SEM. Scale bars, 50 μm (B, D) and 5 μm (B′). Images are shown as maximum intensity projections of Z-stacks encompassing the spinal cord region (B, B′, D: n = 10 slices, step size = 1 μm).

**Suppl. Fig. 3** **Neutrophil ablation does not alter locomotor activity in unlesioned larvae.**

**A:** A schematic timeline of assessing motor activity in unlesioned larvae for (B, C) is shown. **B, C:** Representative swimming behavior tracks of larvae at 4 and 5 dpf are shown (B). Neutrophil ablation does not affect locomotor activity in unlesioned animals at either 4 or 5 dpf. (Two-way ANOVA, interaction (*F* (1,188) = 0.2306, *p* = 0.6316), time (*F* (1,188) = 10.38, *p* = 0.0015), and treatment (*F* (1,188) = 3.422, *p* = 0.0659). Tukey's multiple comparisons test: DMSO (4 dpf vs. 5 dpf), **p* = 0.0467). Error bars show SEM.

**Suppl. Fig. 4 Inhibition of hydrogen peroxide production reduces neutrophil recruitment and impairs axonal regeneration after spinal cord injury.**

**A:** A schematic timeline of DPI treatment for (B–E) is shown. **B, C:** DPI treatment significantly reduces *mpx:gfp^+^* neutrophil numbers at 4 hpl but not at 24 hpl compared with DMSO controls. Dashed boxes indicating the quantification area. (Two-way ANOVA, interaction (*F* (1,48) = 3.224, *p* = 0.0789), time (*F* (1,48) = 26.34, *p* < 0.0001), and treatment (*F* (1,48) = 18.61, *p* < 0.0001). Tukey’s multiple comparisons test: *****p* < 0.0001; ns indicates no significance). **D, E:** DPI treatment does not alter *mpeg1:mCherry^+^* macrophage numbers at 4 and 24 hpl compared with DMSO controls. (Two-way ANOVA, interaction (*F* (1,34) = 0.01227, *p* = 0.9124), time (*F* (1,34) = 170.3, *p* < 0.0001), and treatment (*F* (1,34) = 0.9169, *p* = 0.3451). Tukey’s multiple comparisons test: *****p* < 0.0001; ns indicates no significance). **F:** A schematic timeline of axonal regeneration analysis for (G, H) is shown. **G, H:** DPI treatment significantly reduces axonal bridge thickness compared with DMSO controls at 24 and 48 hpl. (Two-tailed unpaired t-test: G: **p* = 0.0027. H: **p* = 0.0178). Error bars show SEM. Scale bar, 100 μm (B, D), 50 μm (G). Images are shown as maximum intensity projections of Z-stacks encompassing the spinal cord region (B, D, G, H: n = 35 slices, step size = 1 μm).

**Suppl. Fig. 5 MTZ treatment does not affect lesion-induced *il1b* expression in wild type larvae.**

**A:** A schematic timeline of MTZ treatment for B is shown. **B:** A qRT-PCR analysis at 24 hpl shows that MTZ treatment does not significantly alter lesion-induced *il1b* expression in WT larvae compared to DMSO controls. (Two-tailed unpaired t-test, *p* = 0.3569, ns indicates no significance). Error bars show SEM.

**Suppl. Fig. 6 YVAD treatment modulates cytokine expression following lesion in wild-type larvae.**

**A:** A schematic timeline of YVAD treatment (50 µM) for (B-D) is shown. **B:** qRT-PCR analysis at 24 hpl shows that YVAD treatment reduces lesion-induced *il1b* and *tnfa* expression and increases *tgfb1a* expression compared to DMSO controls in WT animals. (Two-tailed unpaired t-test, *il1b*, **p* = 0.0176; *tnfa*, **p* = 0.0464; *tgfb1a*, ***p* = 0.0052). **C, D:** YVAD treatment reduces *il1b* mRNA signal intensity detected by HCR at 24 hpl in the lesioned area in WT animals. (Two-tailed unpaired t-test, **p* = 0.0202). Error bars show SEM. Scale bar, 50 μm (C). Images are shown as maximum intensity projections of Z-stacks encompassing the spinal cord region (C: n = 5 slices, step size = 1 μm).

**Suppl. Fig. 7 Somatic knockout of *il1b* fails to rescue regeneration impairment caused by neutrophil ablation.**

**A:** A schematic diagram of *il1b* gRNAs injection for (B, C) is shown. **B:** RFLP analysis shows disruption of genomic DNA after injection of *il1b* gRNAs targeting exon 3 and 5, respectively. Each lane represents one embryo at 3 dpf. Larvae injected with specific gRNA show digestion-resistant bands (red arrows). Full gel pictures are in supplemental file Tian_et_al_full_gels_RFLP.docx. **C:** A schematic timeline of axonal regeneration analysis for (D, E) is shown. **D, E:** Somatic knockout of *il1b* does not rescue the impaired axonal regeneration caused by neutrophil ablation at 24 hpl, as revealed by anti-acetylated tubulin labelling, compared to controls. (Two-way ANOVA, interaction (F (1,188) = 1.885, *p* = 0.1714), *il1b* gRNAs (F (1,188) = 6.267, *p* = 0.0132), and MTZ treatment (F (1,188) = 6.619, *p* = 0.0109)). Tukey’s multiple comparisons test: *il1b* gRNAs^-^, MTZ^-^ vs. *il1b* gRNAs^-^, MTZ^+^ (**p* = 0.0360); *il1b* gRNAs^-^, MTZ^-^ vs. *il1b* gRNAs^+^, MTZ^-^ (**p* = 0.0261); *il1b* gRNAs*^-^*, MTZ^-^ vs. *il1b* gRNAs^+^, MTZ^+^ (***p* = 0.0010); ns indicates no significance). Error bars show SEM. Scale bar, 50 μm (D). Images are shown as maximum intensity projections of Z-stacks encompassing the spinal cord region (D: n = 35 slices, step size = 1 μm).

**Suppl. Fig. 8 Single-cell and HCR analyses reveal cytokine expression dynamics following spinal cord lesion.**

**A:** t-SEN plot showing major cell populations identified from previously published single-cell RNA-sequencing data (Cavone *et al.*, 2021). **B:** Feature plots showing the expression of *il1b* and *il4* in unlesioned and lesioned conditions at 24 hpl. *Il1b* expression is mainly enriched in macrophages and neutrophils, whereas *il4* expression is detected in neutrophils and enriched in lesioned condition. **C, D:** Representative HCR images showing *il1b* expression (red) together with neutrophils (*mpx:gfp^+^*, green) and macrophages (*mpeg1:mCherry^+^*, magenta) at different time points. *Il1b* expression is primarily associated with neutrophils at 6 hpl, and shifts toward macrophages at 24 and 48 hpl. (Two-way ANOVA, interaction (F (3,74) = 377.2, *p* < 0.0001), time (F (3,74) = 315.1, *p* < 0.0001), and cell type (F (1,74) = 37.22, *p* < 0.0001). Tukey’s multiple comparisons test, *mpeg1:mCherry^+^* labeled with *il1b^+^* vs. *mpx:gfp^+^* labeled with *il1b^+^*, *p* = 0.7257 at 0 hpl, *****p* < 0.0010 at 6, 24 and 48 hpl, ns indicates no significance). Error bar show SEM. Scale bar, 50 μm (C). Images are shown as maximum intensity projections of Z-stacks encompassing the spinal cord region (C: n = 5 slices, step size = 1 μm).

**Suppl. Fig. 9 Neutrophil ablation transiently reduces *il4* mRNA expression.**

**A:** A schematic timeline of qRT-PCR analysis after neutrophil ablation for (B) is shown. **B:** Neutrophils ablation reduces the *il4* expression at 3 dpf, but is not detectably changed after lesion. (Two-tailed unpaired t-test: ***p* = 0.0161 at 3 dpl, *p* = 0.0722 at 4 hpl, ns indicates no significance). **C:** A schematic timeline of *il4* expression analysis by HCR after neutrophil ablation for (E–H). **D, E:** Neutrophil ablation significantly reduces the number of *il4*-expressing neutrophils (*il4*⁺, *mpx:gfp*⁺; double-labeled) at 6 hpl. (Two-way ANOVA, interaction (*F* (2,107) = 68.67, *p* < 0.0001), treatment (*F* (1,107) = 332.2, *p* < 0.0001), and cell category (*F* (2,107) = 124.3, *p* < 0.0001). Tukey’s multiple comparisons test: *****p* < 0.0001. ns indicates no significance). **F, G:** No significant difference is observed in the number of *il4*-expressing neutrophils (*il4*⁺, *mpx:gfp*⁺; double-labeled) following neutrophil ablation at 24 hpl. (Two-way ANOVA, interaction (*F* (2,51) = 4.013, *p* = 0.0241), treatment (*F* (1,51) = 5.907, *p* = 0.0186), and cell category (*F* (2,51) = 120.3, *p* < 0.0001). Tukey’s multiple comparisons test: ***p* = 0.0071. ns indicates no significance). Arrowheads indicate *il4*⁺ neutrophils (*il4*⁺, *mpx:gfp*⁺). Scale bars, 50 μm (D, F). Error bars show SEM. Images are shown as maximum intensity projections of Z-stacks encompassing the spinal cord region (D, F: n = 5 slices, step size = 1 μm).

**Suppl. Fig. 10 *il4*-positive cells that do not label with the *mpx:gfp* transgene are observed in the CHT region before and after lesion.**

**A:** A schematic timeline of the observation of *il4* expression by HCR before and after spinal cord lesion for B is shown. **B:** *il4*-positive cells (magenta) that do not co-localize with *mpx:gfp* (green) are observed (arrowheads) in the CHT of unlesioned and lesioned animals. Scale bar, 100 μm. Images are shown as maximum intensity projections of Z-stacks encompassing the CHT region (B: n = 10 slices, step size = 1 μm).

**Suppl. Fig. 11 Somatic mutation of il4 is effective but does not detectably affect immune cell development and larval growth.**

**A:** RFLP analysis shows effective disruption of genomic DNA after *il4* gRNA injection by resistance to restriction digestion site of the targeted region (red arrows). Each lane represents one larva at 3 dpf. Full gel pictures are in supplemental file Tian_et_al_full_gels_RFLP.docx. **B:** A qRT-PCR analysis shows *il4* mRNA expression is reduced by *il4 gRNA* injection compared to controls at multiple time points. (Two-tailed unpaired t-test: ***p* = 0.0078 at 3 dpf, **p* = 0.0238 at 4 hpl, ****p* = 0.0005 at 24 hpl, ****p* = 0.0001 at 48 hpl). **C:** A schematic timeline of morphological analysis in *il4 gRNA* injected larvae, corresponding to (D–J), is shown. **D, E, F:** No difference of the developmental number of neutrophils (*mpx:gfp*, green) and macrophages (*mpeg1:mCherry*, magenta) was observed in the caudal hematopoietic tissue (CHT) between gControl- and *il4* gRNA-injected larvae at 3 dpf. (Two-tailed unpaired t-test, ns indicates no significance). **G:** A qRT-PCR analysis indicates that *il1b, tnfa*, *tgfb1a* and *tgfb3* expression at 3 dpf are not affect by *il4* gRNA injection, compared to gControl group. (Two-tailed unpaired t-test, ns indicates no significance). **H-J:** Overall morphology of gControl- and *il4 gRNA*-injected larvae reveals no difference in body length (E) or eye diameter (F) at 5 dpf. (Two-tailed unpaired t-test, ns indicates no significance). Error bars show SEM. Scale bar, 100 μm (D), 500 μm (H). Images are shown as maximum intensity projections of Z-stacks encompassing the CHT region (D: n = 35 slices, step size = 1 μm).

**Suppl. Fig. 12 Disruption of il4 delays axon regrowth and resolution of inflammation.**

**A:** A schematic timeline of axonal regeneration analysis for (B–D) is shown. **B, C, D:** *Il4* gRNA injection reduces thickness of the axonal bridge at 24 hpl (C), but not at 48 hpl (D). (Two-tailed unpaired t-test: C: ***p* = 0.0058, D: ns indicates no significance). **E:** A schematic timeline of the number of immune cells analysis for (F–H) is shown. **F-H:** *Il4 gRNA* injection slightly increases neutrophil (*mpx:gfp*, green) numbers and macrophage (*mpeg1:mCherry*, magenta) numbers at 48 hpl, compared to gControl larvae. Dashed boxes indicate the quantification area. (G: Two-way ANOVA, interaction (*F* (2,74) = 4.225, *p* = 0.0183), time (*F* (2,74) = 114.7, *p* < 0.0001), and treatment (*F* (1,74) = 9.283, *p* = 0.0032). Tukey’s multiple comparisons test: **p* = 0.0254; H: Two-way ANOVA, interaction (*F* (2,71) = 1.996, *p* = 0.1434), time (*F* (2,71) = 22.95, *p* < 0.0001), and treatment (*F* (1,71) = 16.56, *p* = 0.0001). Tukey’s multiple comparisons test: ***p* = 0.0015. ns indicates no significance). Error bars show SEM. Scale bar, 50 μm (B), 100 μm (F). Images are shown as maximum intensity projections of Z-stacks encompassing the spinal cord region (B, F: n = 35 slices, step size = 1 μm).

**Suppl. Fig. 13 Heat shock–induced il4 expression increases il4 mRNA levels in neutrophil-ablated larvae.**

**A:** A schematic indicating the *hsp70:il4* plasmid construct and experimental timeline for (B-G) is shown. **B:** A qRT-PCR analysis reveals that heat shock–induced *il4* overexpression in *hsp70:il4* injected larvae strongly increases *il4* mRNA expression at 24 hpl, compared to controls. (One-way ANOVA, *p* = 0.0271. Tukey’s multiple comparisons test: DMSO vs MTZ *hsp70:il4* (**p* = 0.0433), MTZ vs MTZ *hsp70:il4* (**p* = 0.0437), ns indicates no significance). **C-F:** Heat shock–induced *il4* expression increases *il4* mRNA signal intensity in neutrophil-ablated larvae with *hsp70:il4* plasmid at 4 hpl (C) and 24 hpl (E; Two-tailed unpaired t-test: ****p* = 0.0009 (D), ***p* = 0.004 (F)). **G:** Heat-shock-induced over-expression of il4 ( > 30-fold) did not affect il1b expression levels in lesioned non-MTZ treated animals at 24 hpl in the same animals. (Two-tailed unpaired t-test in *il1b*, *p* = 0.8247, ns indicates no significance). Scale bar, 50 μm. Error bars show SEM. Images are shown as maximum intensity projections of Z-stacks encompassing the spinal cord region (D, F: n = 5 slices, step size = 1 μm).

**Supplementary tables**

| **List of targeted genes using CRISPR/Cas9** | | | |
| --- | --- | --- | --- |
| **Gene** | **RE** | **Sequence (5'-3')** | **Primers (5'-3')** |
| *il4* | AvaI | CTTCATTGTGCATTCCCCCG | F: ATGTTCTTCTCTCCACAGACTGG |
|  |  |  | R: AATGGCAGCATGCTTTGGTTTTT |
| *il1b*  (exon3) | BsrDI | CTGTTCAGATCCGCTTGCAA | F: TTGTGGGAGACAGACAGTGC |
|  |  |  | R: AGTCATAAGCAATGCATATCGCT |
| *il1b*  (exon5) | Tsp45I | GCTTCCGGCTCTCAGTGTGA | F: GGTGGATTCAGTGCCGTCTT |
|  |  |  | R: CTTTCAAGTCGCTGCTTCCG |

**Supplementary Table 1 List of targeted genes using CRISPR/Cas9**

|  | | **Primers use for cloning the *hsp70l:il4-t2a-mcherry* vector** | |
| --- | --- | --- | --- |
|  | RE | | Primer sequence (5’-3’) |
| *il4* | FseI | | F: atatggccggccCATGAGGACTTTCTTGCTGTTGG |
|  | AvrII | | R: atatcctaggTGTCCTTTGAGCCGAGTATAG |

**Supplementary Table 2 Primers use for cloning the *hsp70l:il4-t2a-mcherry* vector**

| **qPCR gene list** | **Primer sequence (5’-3’)** |
| --- | --- |
| *il4* | F: CTGTTGGTACTTACATTGGTCCCC |
|  | R: AGTGTCCTGTCTCATATATGTCAGGT |
| *il4r.1* | F: CCACAAAGGTGGCAAACACT |
|  | R: GCGCTTACAATCCAGTGGTG |
| *il1b* | F: GCACATCAAACCCCAATCCAC |
|  | R: GGCACTGAATCCACCACGTT |
| *tnfa* | F: ACAAGATGGAAGTGTGCTGAGA |
|  | R: ATTTCAAGCCACCTGAAGAAAA |
| *tgfb1a* | F: TACGTGGGAAGGCAACACAA |
|  | R: CCCGACTGAGAAATCGAGCC |
| *tgfb3* | F: TGGATTCGGCAAGGACAGAC |
|  | R: ATGTGATTCCCTTCGGGCAG |
| *β-actin* | F: GTGCCCATCTACGAGGGTTA |
|  | R: TCTCAGCTGTGGTGGTGAAG |
| **PCR gene list** | **Primer sequence (5’-3’)** |
| *il4* (primer1) | F: CTGTTGGTACTTACATTGGTCCCC |
|  | R: AGTGTCCTGTCTCATATATGTCAGGT |
| *il4* (primer2) | F: CTTACCCCGGTATATGCTGCA |
|  | R: GGATCCTGAATGGGAAAGGGG |

**Supplementary Table 3 qPCR and PCR primer list**
